# Efficient coding of natural scenes improves neural system identification

**DOI:** 10.1371/journal.pcbi.1011037

**Published:** 2023-04-24

**Authors:** Yongrong Qiu, David A. Klindt, Klaudia P. Szatko, Dominic Gonschorek, Larissa Hoefling, Timm Schubert, Laura Busse, Matthias Bethge, Thomas Euler

**Affiliations:** 1 Institute for Ophthalmic Research, U Tübingen, Tübingen, Germany; 2 Centre for Integrative Neuroscience (CIN), U Tübingen, Tübingen, Germany; 3 Graduate Training Centre of Neuroscience (GTC), International Max Planck Research School, U Tübingen, Tübingen, Germany; 4 Department of Mathematical Sciences, Norwegian University of Science and Technology, Trondheim, Norway; 5 Bernstein Center for Computational Neuroscience, Tübingen, Germany; 6 Research Training Group 2381, U Tübingen, Tübingen, Germany; 7 Division of Neurobiology, Faculty of Biology, LMU Munich, Planegg-Martinsried, Germany; 8 Bernstein Center for Computational Neuroscience, Planegg-Martinsried, Germany; 9 Institute for Theoretical Physics, U Tübingen, Tübingen, Germany; University of Giessen, GERMANY

## Abstract

*Neural system identification* aims at learning the response function of neurons to arbitrary stimuli using experimentally recorded data, but typically does not leverage normative principles such as efficient coding of natural environments. Visual systems, however, have evolved to efficiently process input from the natural environment. Here, we present a normative network regularization for system identification models by incorporating, as a regularizer, the *efficient coding* hypothesis, which states that neural response properties of sensory representations are strongly shaped by the need to preserve most of the stimulus information with limited resources. Using this approach, we explored if a system identification model can be improved by sharing its convolutional filters with those of an autoencoder which aims to efficiently encode natural stimuli. To this end, we built a hybrid model to predict the responses of retinal neurons to noise stimuli. This approach did not only yield a higher performance than the “stand-alone” system identification model, it also produced more biologically plausible filters, meaning that they more closely resembled neural representation in early visual systems. We found these results applied to retinal responses to different artificial stimuli and across model architectures. Moreover, our normatively regularized model performed particularly well in predicting responses of direction-of-motion sensitive retinal neurons. The benefit of natural scene statistics became marginal, however, for predicting the responses to natural movies. In summary, our results indicate that efficiently encoding environmental inputs can improve system identification models, at least for noise stimuli, and point to the benefit of probing the visual system with naturalistic stimuli.

## Author summary

Computational models use experimental data to learn stimulus-response functions of neurons, but they are rarely informed by normative coding principles, such as the idea that sensory neural systems have evolved to efficiently process natural stimuli. We here introduce a novel method to incorporate natural scene statistics to predict responses of retinal neurons to visual stimuli. We show that considering efficient representations of natural scenes improves the model’s predictive performance and produces biologically-plausible receptive fields, at least for responses to noise stimuli. Generally, our approach provides a promising framework to test various (normative) coding principles using experimental data for understanding the computations of biological neural networks.

## Introduction

**Fig 1:**
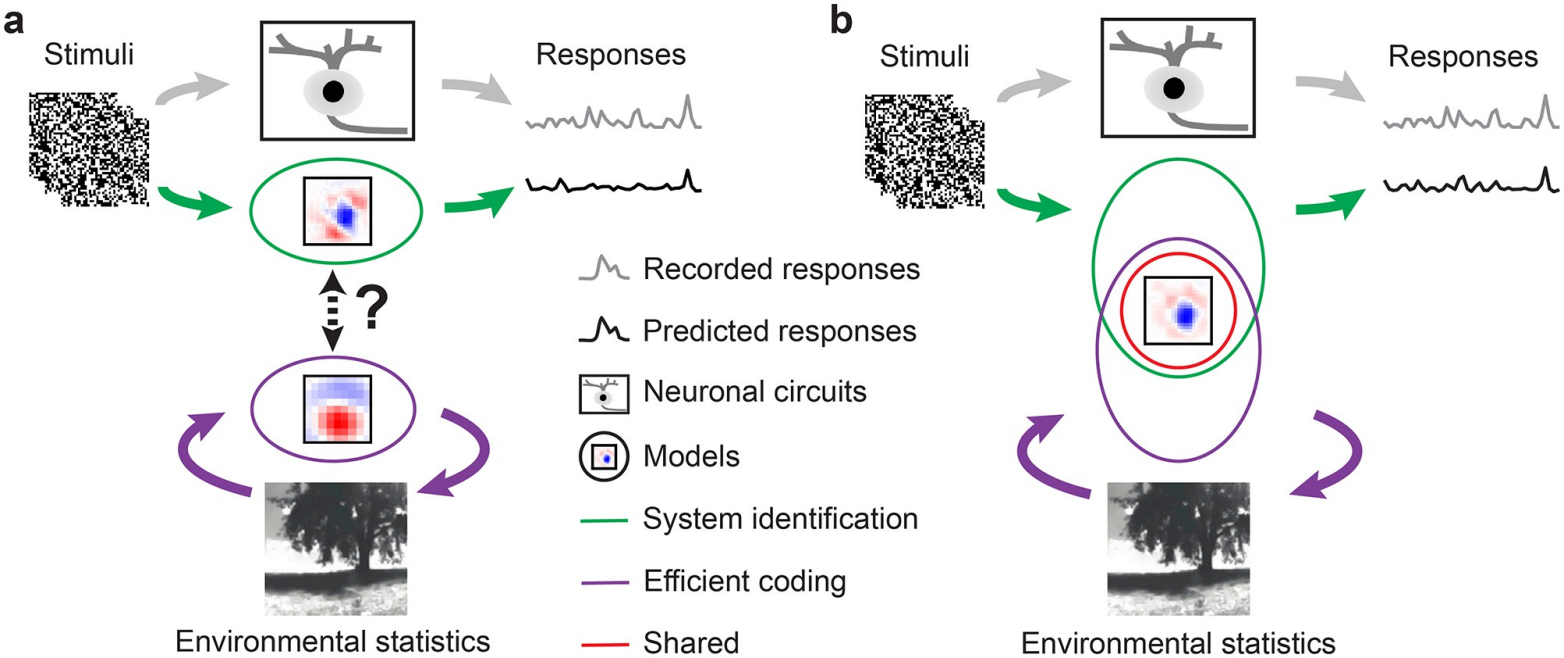
Illustration of our hybrid model combining SI and EC. **a.** Illustration of two common approaches to studying visual systems: system identification, symbolized by the green-labeled branch, aims at predicting responses of neuronal circuits (black rectangle) to specific stimuli, whereas efficient coding (purple-labeled branch) seeks working out principles of the visual system based on environmental statistics. As these two approaches are rarely combined in a single modeling framework, their potential synergies remain largely unexplored. **b.** Our hybrid modeling approach combines system identification (green) and efficient coding (purple) in a single model with shared filters (red circle) to predict neural responses to arbitrary visual stimuli.

In the past years, advances in experimental techniques enabled detailed, large-scale measurements of activity at many levels of sensory processing [1]. As a consequence, *neural system identification* (SI) approaches have flourished (Fig 1a top). They empirically fit the stimulus-response (transfer) function of neurons based on experimentally recorded data [2–4]. A classic example is the generalized linear model (GLM, [2, 5]), which consists of a linear filter as a first order approximation of a neuron’s response function (i.e., its receptive field; [6]), followed by a point-wise nonlinear function for the neuron’s output. To account for additional non-linearities (e.g., [7, 8]), several extensions, such as linear-nonlinear cascades [9, 10], have been proposed. More recently, deep neural network-based SI approaches inspired by the hierarchical processing along the visual pathway [11, 12] have been developed (reviewed in [13–17]). While SI methods became particularly successful in predicting responses of visual neurons [18–22], they often require large amounts of training data and, more critically, do rarely consider adaptions to the natural environment.

However, like other senses, vision has evolved to promote a species’ survival in its natural environment [23], which is thought to have driven visual circuits to efficiently represent information under a number of constraints, including metabolic limits and space restrictions [24, 25]. As a consequence, the visual system has adapted to natural statistics, as shown, for example, by the fact that the distribution of orientation preferences of visual neurons mirrors the dominance of cardinal orientations in natural scenes [26–28].

Such adaptations are at the heart of *efficient coding* (EC) approaches (Fig 1a bottom): They derive algorithmic principles underlying neural systems from the statistical properties of natural stimuli and by incorporating biological constraints [15, 24, 25, 29–31]. Here, one popular strategy starts from the assumption that early visual processing serves to decorrelate the redundant signals in natural environments [32–34]. This theory can reproduce feature selectivity, e.g., difference-of-Gaussian (DoG) kernels that have similar receptive field (RF) properties as retinal ganglion cells (RGCs) [35]. Recently, deep neural network-augmented EC approaches were proposed, such as convolutional autoencoders [36, 37], which are trained to optimally reconstruct inputs in the presence of an information “bottleneck” (i.e., from a constrained latent representation). Such convolutional autoencoders have been shown to yield center-surround spatial RFs with similar properties as those observed in RGCs when encoding either pink (1/*f*) noise or natural scenes [38, 39]. A downside of EC is that it is not always straightforward to experimentally measure coding efficiency and feature selectivity predicted by these approaches in neural systems (discussed in [40, 41]) and, hence, the interpretation of EC models with respect to the biological underpinnings remains challenging.

Notably, the intersection between EC and SI has long remained largely unexplored but lately shifted into focus. For instance, Młynarski and colleagues recently proposed a theoretical framework incorporating normative theories for statistical inference on simulated or pre-fit neural data [42]. Their framework enables conducting rigorous statistical hypothesis tests of coding principles, but has not yet been applied to predicting neural responses to arbitrary stimuli with minimal assumptions.

Here, we tested whether the EC hypothesis can serve as a useful regularization for learning the response functions of neurons. To do so, we built a hybrid model combining a SI branch with an EC branch, forced the two branches to share filters (Fig 1b), and asked if knowledge about natural scene statistics could help predicting retinal responses. To this end, we experimentally recorded Ca^2+^ signals of neurons in the mouse retina while presenting it with noise stimuli. We then used the responses to train the SI branch, which aimed to predict retinal responses. We used natural movies that we recorded in mouse habitats outdoors to train the EC branch, which aimed to represent natural scenes efficiently [39]. We found a synergy between neural prediction and natural scene statistics: First, for noise stimuli, the hybrid approach had a better predictive performance than a pure SI approach. Second, compared to the SI model, the hybrid model produced filters with a clearer center-surround RF structure, akin to RFs at early visual processing stage. However, we did not observe such a synergy for the prediction of responses to natural movies. Our results demonstrate that predicting sensory responses, in particular to noise stimuli, benefits from considering adaptations to the natural environment, and thus highlights the benefits of naturalistic stimuli for vision research.

## Materials and methods

### Ethics statement

All procedures were performed in accordance with the law on animal protection issued by the German Federal Government (Tierschutzgesetz) and approved by the institutional animal welfare committee of the University of Tübingen.

### Animal procedures and retinal activity recordings

#### Animal procedures

We used n = 5, 5–9 weeks old female C57BL/6 mice (wild-type; JAX 000664, Jackson Laboratory, USA). Due to the exploratory nature of our study, we did not use any statistical methods to predetermine sample size, nor did we perform blinding or randomization. Animals were housed under a standard light-dark (12h:12h) cycle. All procedures were carried out under very dim red illumination (>650 nm). Prior to the start of the experiment, animals were dark-adapted for ≥1 h, then anesthetized with isoflurane (Baxter, Germany), and killed by cervical dislocation.

The eyes were enucleated and hemisected in carboxygenated (95% O_2_, 5% CO_2_) artificial cerebrospinal fluid (ACSF) solution containing (in mM): 125 NaCl, 2.5 KCl, 2 CaCl_2_, 1 MgCl_2_, 1.25 NaH_2_PO_4_, 26 NaHCO_3_, 20 glucose, and 0.5 l-glutamine (pH 7.4). Next, the retina was flat-mounted onto an Anodisc (#13, 0.1 *μm* pore size, GE Healthcare, Germany) with the ganglion cell layer (GCL) facing up. To uniformly label the GCL cells, bulk electroporation was performed with the fluorescent Ca^2+^ indicator Oregon-Green BAPTA-1 (OGB-1; Invitrogen, Germany), as described earlier [43, 44], using 4-mm plate electrodes (CUY700P4E/L, Xceltis, Germany) and 9 pulses (∼9.2 V, 100 ms pulse width at 1 Hz). After electroporation, the tissue was immediately moved to the microscope’s recording chamber, where it was continuously perfused with carboxygenated ACSF at ∼36°C and left to recover for ∼30 min before recordings started. Additionally, Sulforhodamine-101 (SR101, Invitrogen, Germany) was added to the ACSF (∼0.1 *μ*M final concentration) to visualize blood vessels and identify damaged cells.

#### Two-photon Ca^2+^ recordings and light stimulation

We recorded light stimulus-evoked Ca^2+^ signals in GCL cells of the explanted mouse retina using a MOM-type two-photon (2P) microscope [45, 46] from Sutter Instruments (purchased from Science Products, Germany), as described earlier [44, 47]. In brief, the microscope was powered by a mode-locked Ti: Sapphire laser (MaiTai-HP DeepSee, Newport Spectra-Physics, Germany) at 927 nm. Two detection pathways allowed simultaneously recording of OGB-1 and SR101 fluorescence (HQ 510/84 and HQ 630/60, respectively; both Chroma/AHF, Germany) through a 16x water immersion objective (CFI75 LWD16 /0.8W, DIC N2, Nikon, Germany). A custom-written software (ScanM, by M. Müller and T.E.) running under IGOR Pro 6.3 for Windows (Wavemetrics, USA) was used to acquire time-lapsed (64x64 pixels) image scans at a frame rate of 7.8125 Hz. Higher resolution images were acquired using 512x512 pixel scans. Additionally, to register the scan field positions, the outline of the retina and the optic disc were traced.

The retinas were presented with color noise stimulus using a visual stimulator tuned to the spectral sensitivities of mice [48]. This stimulus consisted of independent binary dense noise (28x28 pixel frames, each pixel covering (0.83°)^2^ of visual angle) in the UV and green stimulator channels at 5 or 30 Hz. The stimulus contained 5 different training sequences (96 s each) interspersed with 6 repeats of a 10 s test sequence (S1(a) Fig).

In total, we used four data sets for modeling: (*i*) responses of n = 96 GCL neurons to 5-Hz noise recorded in dorsal retina (n = 2 eyes); (*ii*) responses of n = 427 GCL neurons to 5-Hz noise recorded ventrally (n = 5 eyes); in this dataset, we also presented two other stimuli: a full-field chirp (700 *μ*m in diameter) and a moving bar stimulus (300x1,000 *μ*m bright bar moving at 8 directions at 1 mm/s). The responses to these latter stimuli were used to functionally classify the recorded GCL neurons [47]. (*iii*) n = 64 GCL neurons to 30-Hz noise recorded ventrally (n = 2 eyes). (*iv*) n = 86 GCL neurons to 30-Hz natural movie recorded ventrally (n = 1 eye). All cell numbers are after quality control (see below).

#### Data preprocessing and analysis

For each cell, we calculated a quality index (*QI*, with 0 ≤ *QI* ≤ 1) for its responses to each stimulus type as follows:

$$\begin{array}{c} QI=\text{Var}{\left[E{\left[C\right]}_{r}\right]}_{t}/E{\left[\text{Var}{\left[C\right]}_{t}\right]}_{r} \end{array}$$ (1)

where C is a t-by-r response matrix (time samples, *t*, by repetitions, *r*). The higher *QI*, the more reliable the response and the higher the signal-to-noise ratio. For the noise stimulus, *QI*_*noise*_ was determined based on the test sequence responses. For the following analysis, we only used cells with *QI*_*noise*_ > 0.25; in case chirp and moving bar responses were also recorded, neurons had to fulfill *QI*_*chirp*_ > 0.35 or *QI*_*bar*_ > 0.6 to be included.

In case of the noise stimulus, we preprocessed each cell’s Ca^2+^ signal by Z-normalizing the raw traces and matching sampling frequency of the recording (7.8125 Hz) to the stimulus frequency (5 or 30 Hz) via linear interpolation. Then, the traces were detrended using a high-pass filter (> 0.1 Hz) and their 1^st^ order derivatives were calculated, with negative values set to zero. We used the average of a cell’s responses to the 6 test sequence repeats as ground truth. Excluding the test sequences, we had per cell a total of 480 s of data, of which we used 440 s (∼91%) for training and the remaining 40 s (∼9%) for validation (i.e., to pick the hyperparameters of the SI model, see below).

For the responses to the natural movie stimulus, we used the average of a cell’s responses to the 3 test sequence repeats as ground truth. Excluding the test sequences, we had per cell a total of 540 s of data, of which we used 433 s for training and the remaining 107 s for validation. Note that as input for the models, we down-sampled the natural movie stimulus to 36x32 pixel frames to match it to the resolution of the noise stimulus.

For chirp and moving bar responses, we first detrended the traces and then normalized them to [0, 1] [44]. Using these responses, the cells were classified to different functional groups [47] using RGC type classifier (see below).

To estimate the directional tuning from the moving bar responses, we first performed singular value decomposition (SVD) on the mean response matrix, resulting in a temporal and a directional component. We then summed the directional vectors in 2D planes and used the resulting vector length as direction selectivity index. Next, by shuffling trial labels and computing the tuning curve for 1,000 times (permutation test), we got the null distribution (no directional tuning). The percentile of true vector length was used as p-value of directional tuning [47]. Here, we considered cells with *p* < 0.05 as direction-selective (DS) and the remaining ones as non-DS.

#### RGC type classifier

To predict the functional type of GCL cells, we used a Random Forest Classifier (RFC; [49]), which was trained on a published mouse dataset [47]. In that study, features were extracted from the responses to different visual stimuli (e.g., chirp and moving bar) and used to cluster GCL cells into 32 RGC types and 14 additional dAC types. Here, we learned a mapping *f* from response features (20 features from responses to chirp, *ϕ*_*chirp*_ and 8 features from responses to moving bar stimulus, *ϕ*_*mb*_) and two additional parameters Θ = {*θ*_*soma*_, *θ*_*DS*_} to functional cell type labels *L* by training a RFC for the dataset from [47]:

$$\begin{array}{c} f:(\phi_{chirp},\phi_{bar},\Theta )\;\mapsto \;L \end{array}$$ (2)

where *θ*_*soma*_ denotes soma size to distinguish between alpha and non-alpha RGC types and *θ*_*DS*_ denotes p-value of permutation test for direction selectivity to distinguish between DS and non-DS RGC types.

We fit the RFC on a subset of data from [47] and validated its performance on a held-out test dataset. The classifier had a prediction accuracy of ∼76% on a held-out test dataset (S5 Fig). To apply the trained classifier to our newly recorded dataset, we projected the RGC responses (normalized to [−1, 1]) into the feature space described in [47] by computing the dot product between the response and the feature matrices. We used the RFC implementation provided by the python package scikit-learn [50] to train the classifier.

### 2D models

#### Stand-alone SI model (2D)

**Fig 2:**
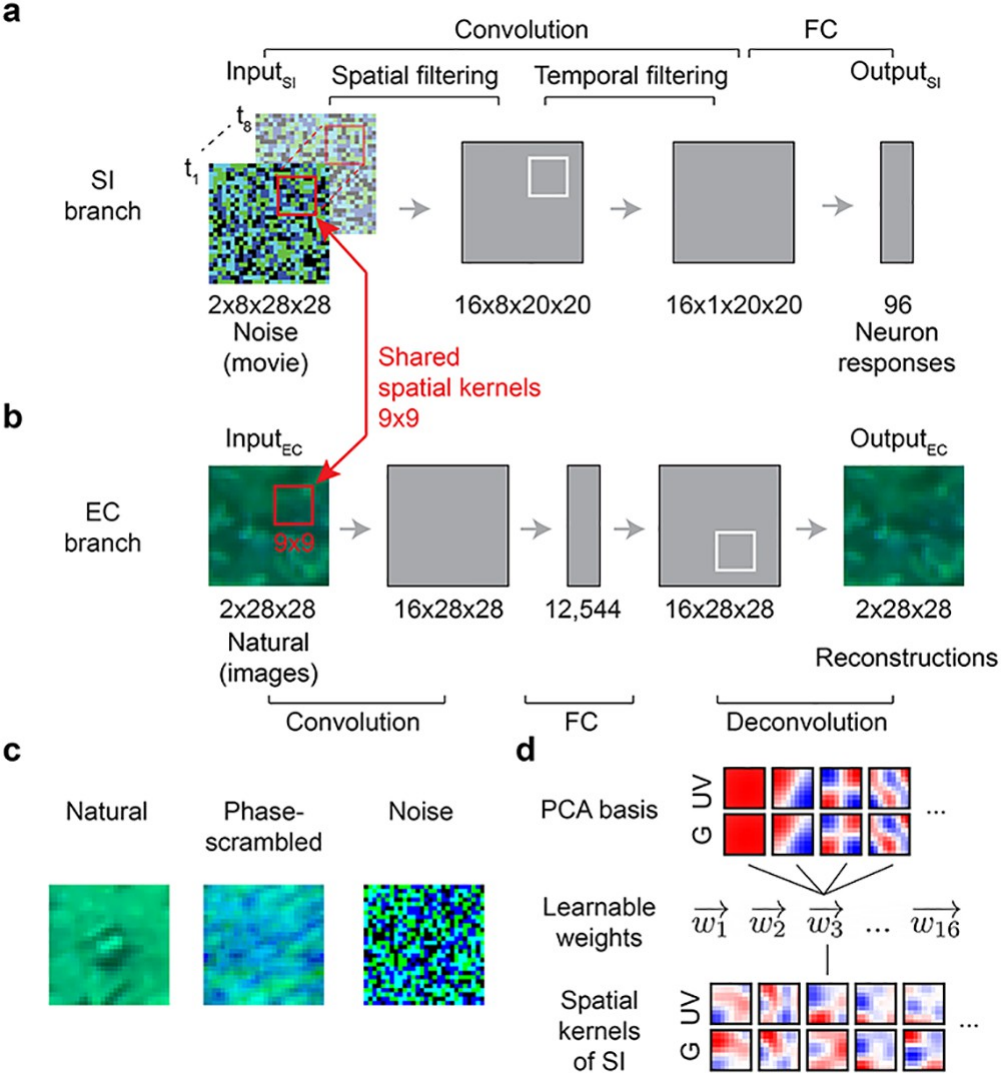
Hybrid model with shared spatial filters. **a,b.** Schemata of SI model (a) and EC model (b) from [39]. The SI model branch consists of spatial and temporal convolutional layers, a fully connected (FC) layer and a nonlinear layer (see Methods). The EC model branch is a convolutional autoencoder, consisting of an encoder and a decoder network. In the hybrid model, the two branches were trained in parallel with shared spatial filters (all spatial filters were shared; red). Input_SI_: 8-frame UV-green noise (*t*_1_ … *t*_8_); Output_SI_: predicted GCL cell Ca^2+^ responses; Input_EC_: UV-green natural images; Output_EC_: reconstructed Input_EC_. **c.** Example for the different inputs (natural images, phase-scrambled natural images, and noise) for the EC branch in hybrid models (*hybrid-natural*, *hybrid-pha-scr*, *hybrid-noise*). **d.** Using PCA filters as basis vectors for spatial convolutional filters of the SI model; *SI-PCA* learned 16 weight vectors ($\vec{w_{1}}\;\ldots \;\vec{w_{16}}$) with same vector length as the number of PCA basis elements.

As baseline model to predict the responses of neurons to the noise stimulus, we employed a stand-alone SI model (supervised learning), in which we used factorized spatial and temporal convolutional filters (cf. Fig 2a; [51, 52]). This SI model consisted of one spatial convolutional layer (16x2x1x9x9, output channels x input channels x depth x image width x image height), one temporal convolutional layer (16x16x8x1x1, with 8 stimulus frames preceding an event for noise; 16x16x50x1x1, with 50 stimulus frames preceding an event for natural movie), and—after flattening the spatial dimension—one fully connected layer (FC; 96x6,400 for noise stimulus, and 86x10,752 for natural movie, output x input channels), followed by an exponential function. No padding was used. We tested different filter channel numbers and found the number = 16, 24, 32 had similar performance (higher than number = 8) on our datasets. Then we picked a relatively small number = 16 as the autoencoder models desired large memory in hidden layers (see below). The loss function was defined as:

$$\begin{array}{c} \begin{array}{c} L_{SI}=\sum_{i}\left(\hat{\vec{r_{i}}}-\vec{r_{i}}log\hat{\vec{r_{i}}}\right)+\alpha_{1}\|\vec{w_{cs}}{\|}_{2}+\alpha_{2}\|\vec{w_{ct}}{\|}_{2}+\beta {\left\|\vec{w_{f}}\right\|}_{1} \end{array} \end{array}$$ (3)

Here, the first term is the Poisson loss between predicted responses ($\hat{\vec{r_{i}}}$) and ground truth ($\vec{r_{i}}$) (with *i* denoting the neuron index), the second term is the L2 penalty on the weights of the spatial convolutional filters ($\vec{w_{cs}}$) with hyperparameter *α*_1_, the third term is the L2 penalty on the weights of temporal convolutional filters ($\vec{w_{ct}}$) with hyperparameter *α*_2_, and the last term is the L1 penalty on the FC layer ($\vec{w_{f}}$) with hyperparameter *β*. We note that, compared to the EC branch of hybrid model, penalty on filter weights could be seen as an implicit form of efficient energy coding, limiting synaptic transmission and generating kernels akin to representations in early visual system ([53]).

After performing a grid search for the three hyperparameters, we picked *α*_1_ = 10, *α*_2_ = 10, *β* = 1/16 which yielded the best performance on the validation data. After training, we estimated the neurons’ spatio-temporal RF filters by computing gradients for each neuron, starting with a blank image sequence as input. These gradients represent the first-order approximation of the input that maximizes the neuron’s activation [6]. For visualization, we extracted the spatial and temporal RFs via SVD.

As a metric of biological plausibility, we calculated the coefficient of determination (R-squared; [0, 1]) of fitting 2D Gaussian distributions to the spatial (component of) the convolutional filters. We set the R-squared value to 0 if the sigma of the fitted Gaussian was larger than the size of the filter (i.e., 9 pixels). We calculated this fit quality for the filter of the chromatic channel with the dominant response. Because the mouse retina is divided into a more green-sensitive dorsal and a more UV-sensitive ventral retina (e.g., [44]), this meant that for dorsal neurons we only determined the R-squared for filters for the green stimulus channel, and for ventral neurons for the UV stimulus channel.

#### SI-PCA model (2D)

The spatial convolutional filters of the SI-PCA model were composed from PCA basis functions (*W*). The model was trained to learn the weights of these basis functions. The filters were produced by performing PCA transformation on natural images recorded in mouse habitats [39]:

$$\begin{array}{c} W=U^{T} \end{array}$$ (4)

where *U* contains the eigenvectors of the covariance matrix of the centered data in each column.

For example, when using 4 PCA bases, the shape of learnable weight matrix was 16x4 (channel number x basis number), the shape of PCA bases was 4x2x1x9x9 (basis number x chromatic channel x depth x image width x image height), and the resulted spatial filter had the shape of 16x2x1x9x9. We varied the number of used basis (hyperparameter) and selected the one which achieved the best performance on validation data (S1(b) and S3(b) Figs).

#### SI-DCT model (2D)

For the SI-DCT model, its spatial convolutional filters were composed from DCT basis functions, which were defined as:

$$\begin{array}{c} F\left(u,v\right)=\alpha \left(u\right)\alpha \left(v\right)\;\text{cos}\left[\frac{(2i+1)\pi }{2N}u\right]\text{cos}\left[\frac{(2j+1)\pi }{2N}v\right] \end{array}$$ (5)

$$\begin{array}{c} \alpha \left(u\right)=\{\begin{array}{cc} \sqrt{\frac{1}{N}} & u=0\\ \sqrt{\frac{2}{N}} & u\neq 0 \end{array} \end{array}$$ (6)

$$\begin{array}{c} \alpha \left(v\right)=\{\begin{array}{cc} \sqrt{\frac{1}{N}} & v=0\\ \sqrt{\frac{2}{N}} & v\neq 0 \end{array} \end{array}$$ (7)

where *i* and *j* denote pixel index of the input image (size (*N*, *N*)); *u* and *v* denote DCT coefficient index of the DCT filter. Here, we employed DCT basis functions for one-channel gray images and thus used different bases for each chromatic channel. For example, when using 4 DCT bases, the shape of learnable weight matrix was 16x4x2 (channel number x basis number x chromatic channel), the shape of basis function was 4x1x9x9 (basis number x depth x image width x image height), and the resulted spatial filter had the shape of 16x2x1x9x9. Like for SI-PCA, we varied the number of used basis and picked the one which achieved the best performance on validation data (S1(b) Fig).

#### Stand-alone EC model (2D)

We used a similar EC model architecture (convolutional autoencoder) and loss function as in [39]. The model’s encoder contained a single convolutional layer (with weights denoted $\vec{w_{c}}$) followed by a rectified linear unit (ReLU) function, one FC layer, and another ReLU function. The decoder contained one FC layer, one ReLU function, a single deconvolutional layer (with weights denoted $\vec{w_{d}}$), and a hyperbolic tangent (tanh) function to map back to the original data range ([−1, 1]).

As a measure of reconstruction quality, we used mean squared error (MSE; [38, 39]). We did not use a classical bottleneck with a limited number of units as encoder output layer. Instead, we added Gaussian noise to the encoder output for redundancy reduction [38, 54, 55] and an L1 penalty (hyperparameter *β*) was imposed to its activation ($\vec{h}$) for sparse readouts [38, 54, 56]. We also applied L2 regularization on the convolutional and deconvolutional layers to encourage the learning of smooth filters [53, 57, 58]. We used 16 9x9 convolutional and deconvolutional filters. The activation tensor (16x28x28, output channel x image width x image height) following the first convolutional layer was flattened to a one-dimensional vector with 12,544 inputs before feeding into the FC layer. The loss function for the EC model was:

$$\begin{array}{c} L_{EC}=\sum_{i}{\left(\vec{x_{i}}-\hat{\vec{x_{i}}}\right)}^{2}+\alpha (\|\vec{w_{c}}{\|}_{2}+\|\vec{w_{d}}{\|}_{2})+\beta \|\vec{h}{\|}_{1} \end{array}$$ (8)

where the first term is the MSE error between the prediction $\hat{\vec{x_{i}}}$ and ground truth $\vec{x_{i}}$ with image index *i*, and the next two terms denote the L2 and L1 penalties. This way, the EC model learns smooth convolutional filters resembling 2D Gaussians, reminiscent of retinal representations [38, 39].

#### Hybrid model (2D)

The hybrid (semi-supervised) model consisted of a SI and an EC branch (for details on the two models’ architectures, see above). These branches were trained simultaneously, sharing the spatial convolutional filters ($\vec{w_{cs}}$). The total loss function of the hybrid model was derived from the loss functions of the two branches as follows:

$$\begin{array}{c} L_{Hybrid}=wL_{SI}+\left(1-w\right)L_{EC} \end{array}$$ (9)

$$\begin{array}{c} \begin{array}{c} L_{SI}=(\sum_{i}\left(\hat{\vec{r_{i}}}-\vec{r_{i}}log\hat{\vec{r_{i}}}\right)+\alpha_{1}\|\vec{w_{cs}}{\|}_{2}+\alpha_{2}\|\vec{w_{ct}}{\|}_{2}/w+\beta_{1}\|\vec{w_{f}}{\|}_{1}/w)/N_{1} \end{array} \end{array}$$ (10)

$$\begin{array}{c} \begin{array}{c} L_{EC}=(\sum_{j}{\left(\vec{x_{j}}-\hat{\vec{x_{j}}}\right)}^{2}+\alpha_{3}\|\vec{w_{cs}}{\|}_{2}+\alpha_{3}\|\vec{w_{d}}{\|}_{2}/\left(1-w\right)+\beta_{2}\|\vec{h}{\|}_{1}/\left(1-w\right))/N_{2} \end{array} \end{array}$$ (11)

Here, *i* and *j* denote neuron and image index, respectively; *N*_1_ and *N*_2_ the number of neurons and images, respectively. The weight (*w*, with 0 ≤ *w* ≤ 1) controlled the impact of each branch’s loss function on the shared spatial filters. Practically, we used *w* = 10^−8^ for *L*_*SI*_ and *w* = (1 − 10^−8^) for *L*_*EC*_ when *w* = 0 and *w* = 1, respectively. Note that we added *w* to the denominator of the last two terms to maintain the same regularization for $\vec{w_{ct}}$ and $\vec{w_{f}}$ in a stand-alone SI model when varying *w*. For *L*_*EC*_, similar to *L*_*SI*_, we added (1−*w*) to the denominator of the last two terms to keep the same regularization for $\vec{w_{d}}$ and $\vec{h}$ in a stand-alone EC model when varying *w*. We used different data to train the EC branch of the hybrid model: natural images, phase-scrambled natural images and noise. All hybrid models were trained for a maximum of 100 epochs (S1(c) and S1(d) Fig); training was stopped early when the prediction on validation data started decreasing.

Tuning all hyperparameters jointly in a grid search was computationally prohibitive. Hence, for the SI branch, we varied the hyperparameters around those determined for the stand-alone configuration (*α*_1_ = 10, *α*_2_ = 10, *β*_1_ = 1/16; see above), while for the EC branch, we varied the hyperparameters systematically around the values (*α*_3_ = 10^3^, *β*_2_ = 1/16) used in [39]. To tune *w*, we devised a linear search approach by normalizing the loss functions (using *N*_1_ and *N*_2_). This way, we were able to compare the pure SI and hybrid models, both with best predictive performance, and ensured the performance difference between them came from the EC regularizer.

**Fig 3:**
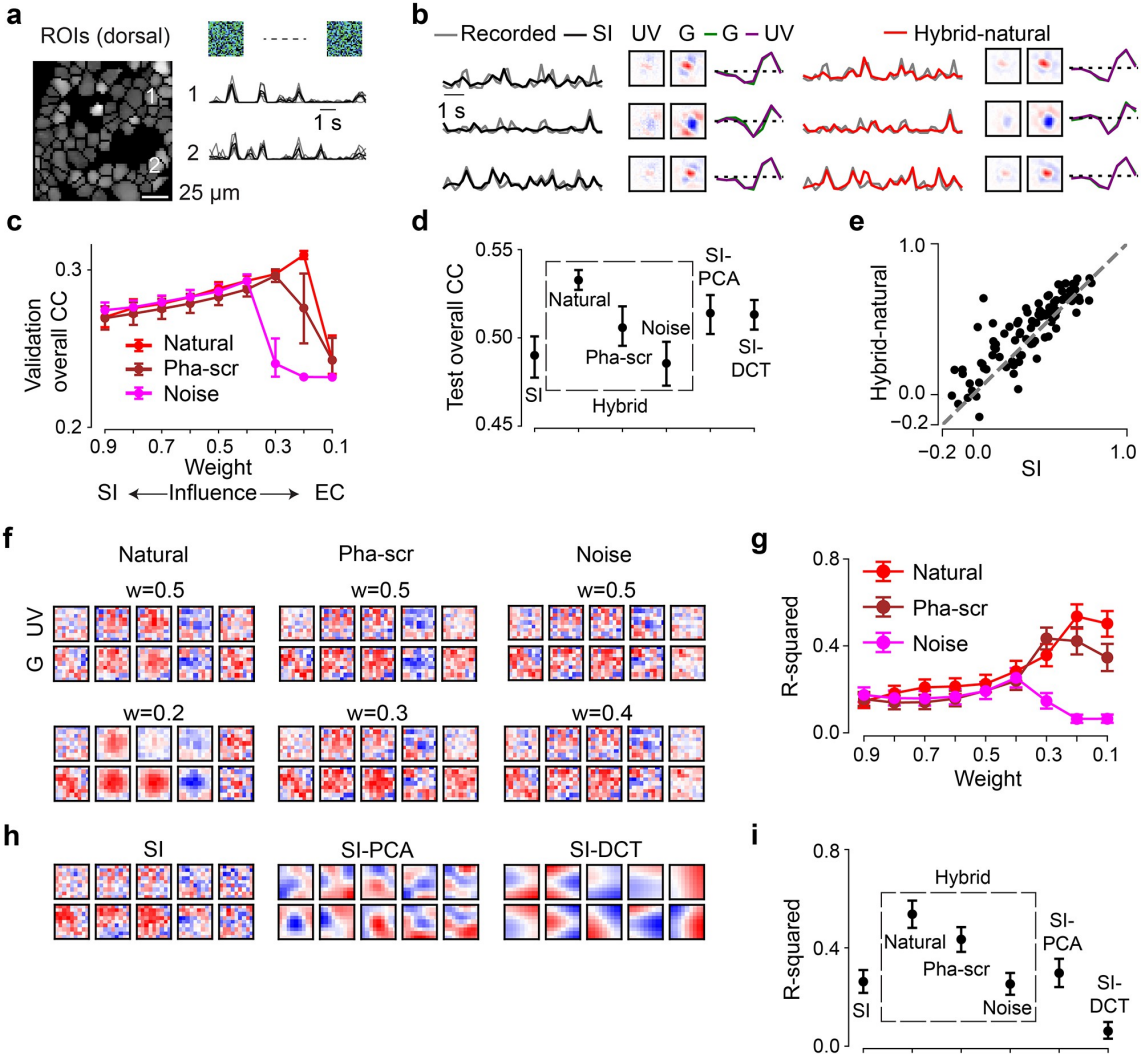
Neural encoding tasks benefit from natural scene statistics. **a.** Region-of-interest (ROI) mask of one recording field in dorsal retina (left) and mean Ca^2+^ responses (black) of exemplary ROIs in response to 6 repeats of noise stimuli (single trials in gray). **b.** Three representative GCL cell responses (gray) to the noise stimulus (cf. Fig 2a, left), together with predictions of best performing models on test data (black, SI; red, hybrid w/ natural scenes as input to the EC path, i.e., Input_EC_), and learned spatio-temporal receptive fields (RFs) visualized by SVD. **c.** Model performance (linear correlation coefficient, CC; mean for *n* = 10 random seeds per model) based on validation data for hybrid model with natural scenes (red), with phase-scrambled scenes (brown), or with noise (magenta) as Input_EC_, and for different weights. Note that the correlation values for the validation data are relatively low because these predictions were calculated on a single-trial basis (Methods). **d.** Best performance (mean for *n* = 10 random seeds per model) based on test data for SI, *SI-PCA* (16 bases), *SI-DCT* (4 bases), *hybrid-natural* (*w* = 0.2), *hybrid-pha-scr* (*w* = 0.3) and *hybrid-noise* (*w* = 0.4; *p* < 0.0001 for SI vs. *hybrid-natural*, *p* = 0.0085 for *SI-PCA* vs. *hybrid-natural*, *p* = 0.0011 for *hybrid-natural* vs. *hybrid-pha-scr*, two-sided permutation test, *n* = 10, 000 repeats). **e.** Scatter plot for model predictions based on test data for *hybrid-natural* (*w* = 0.2) vs. SI at one random seed, with each dot representing one neuron. **f.** Representative spatial filters (shared convolutional filters) for hybrid models with different Input_EC_ and different weights. Upper: with *w* = 0.5; lower: with optimal *w* (see (c)) for hybrid models. **g.** Mean R-squared of fitting a 2D Gaussian to spatial filters (cf. (f)), for hybrid model with natural scenes (red), with phase-scrambled scenes (brown), or with noise (magenta) as Input_EC_, and for different *w* (n = 10 random seeds per model). **h.** Representative spatial filters (shared convolutional filters) for SI, SI with PCA filters (16 bases) and SI with DCT filters (4 bases). **i.** Mean R-squared of fitting a 2D Gaussian to the spatial filters for one chromatic stimulus channel (green; *n* = 10 random seeds per model; *p* < 0.0001 for SI vs. *hybrid-natural*, *p* < 0.0001 for *SI-PCA* vs. *hybrid-natural*, *p* = 0.0074 for *hybrid-natural* vs. *hybrid-pha-scr*, two-sided permutation test, *n* = 10, 000 repeats). Error bars in (c),(d),(g),(i) represent 2.5 and 97.5 percentiles obtained from bootstrapping.

After training the hybrid model, we estimated the spatio-temporal RFs of all neurons using a gradient ascent algorithm [6]. We visualized the spatial and temporal component of RFs using SVD (cf. Fig 3b), and the magnitude of the RF was indicated in the spatial component.

We trained 2D models using all training data (440 s) with a learning rate of *μ* = 10^−4^. In case less data were used (i.e., to evaluate data efficiency), we kept all hyperparameters the same as for the full data case but doubled the learning rate. This was done because the stand-alone SI model and the hybrid model could not reach the minimum of validation loss within 100 epochs (when less data were used).

### 3D models

#### Stand-alone SI model (3D)

The 3D SI model consisted of one spatio-temporal convolutional layer (16x2x8x9x9, output channels x input channels x depth x image width x image height; depth varied with the frequency of noise stimuli, n = 8 and n = 30 for 5-Hz and 30-Hz noise, respectively), and—after flattening all dimension—one FC layer (96x6,400, output channels x input channels; output channel varied with cell numbers n = 96, 64 or 427 for different data sets; see above), followed by an exponential function. No padding was used. The loss function was defined as:

$$\begin{array}{c} L_{SI}=\sum_{i}\left(\hat{\vec{r_{i}}}-\vec{r_{i}}\;log\;\hat{\vec{r_{i}}}\right)+\alpha \|\vec{w_{c}}{\|}_{2}+\beta {\left\|\vec{w_{f}}\right\|}_{1} \end{array}$$ (12)

This equation differs from Eq (3) with respect to the L2 penalty, which is here on the weights of the spatio-temporal convolutional filters ($\vec{w_{c}}$) with hyperparameter *α* for the second term. After performing a grid search for the two hyperparameters, we picked *α* = 100, *β* = 1/4 which yielded the best performance on the validation data. After training, we estimated and extracted the cells’ spatial and temporal RFs via SVD for visualization.

#### SI-PCA model (3D)

For the 3D SI-PCA models, we applied Eq (4) to the movie clips (2x8x9x9, chromatic channel x depth x image width x image height; depth varied with the frequency of noise stimuli, n = 8 and n = 30 for 5-Hz and 30-Hz noise, respectively). Like for 2D SI-PCA models, we varied the number of used bases and picked the number for which the model achieved the best performance on the validation data (S3(a) Fig).

#### Stand-alone EC model (3D)

The 3D EC models used a sequence of frames from a movie clip as input and featured 3D spatio-temporal convolutional layers (with weights denoted $\vec{w_{c}}$) in the encoder. The decoder contained deconvolutional layers with weights $\vec{w_{d}}$. In the past-encoding case, we fed an 8-frame clip (frames at *t* − 7 to *t*) to the model and aimed at reconstructing the 7^th^ frame (at *t* − 1). In the future-prediction case, the goal was to predict the 8^th^ frame (at *t*) with the input being the first 7 frames (*t* − 7 to *t* − 1) of the clip. The loss functions was similar to that given by Eq (8) except that (*i*) $\vec{w_{c}}$ features different a shape (16x2x8x9x9, output channel x chromatic channel x filter depth x filter width x filter height), and (*ii*) *x*_*i*_ denotes the 7^th^ frame for the past encoding and the 8^th^ frame for the future prediction model (S2(b), S2(c) and S2(d) Fig).

#### Hybrid model (3D)

The 3D hybrid models consisted of a SI branch and an EC branch with shared spatio-temporal convolutional filters ($\vec{w_{c}}$; see above). Like for the 2D hybrid models, the total loss function was a weighted sum of losses for the two branches as follows:

$$\begin{array}{c} L_{Hybrid}=wL_{SI}+\left(1-w\right)L_{EC} \end{array}$$ (13)

$$\begin{array}{c} \begin{array}{c} L_{SI}=(\sum_{i}\left(\hat{\vec{r_{i}}}-\vec{r_{i}}\;log\;\hat{\vec{r_{i}}}\right)+\alpha_{1}\|\vec{w_{c}}{\|}_{2}+\beta_{1}\|\vec{w_{f}}{\|}_{1}/w)/N_{1} \end{array} \end{array}$$ (14)

$$\begin{array}{c} \begin{array}{c} L_{EC}=(\sum_{j}{\left(\vec{x_{j}}-\hat{\vec{x_{j}}}\right)}^{2}+\alpha_{2}\|\vec{w_{c}}{\|}_{2}+\alpha_{2}\|\vec{w_{d}}{\|}_{2}/\left(1-w\right)+\beta_{2}\|\vec{h}{\|}_{1}/\left(1-w\right))/N_{2} \end{array} \end{array}$$ (15)

Here, *i* denotes neuron index, *j* movie clip index, *N*_1_ neuron number, and *N*_2_ the number of movie clips. Again, instead of tuning all hyperparameters jointly via a grid search, we varied the hyperparameters around the values determined for the stand-alone SI configuration (*α*_1_ = 100, *β*_1_ = 1/4) for the SI branch. For the EC branch, we varied the hyperparameters systematically around the values (*α*_2_ = 10^4^, *β*_2_ = 1/16) used in the stand-alone EC models. We then tuned *w* linearly after normalizing the loss functions (using *N*_1_ and *N*_2_). We also visualized the spatial and temporal RF components using SVD.

## Results

### Hybrid system identification and efficient coding models

To test if learning an efficient representation of natural input could help predict neuronal responses in the early visual system, we employed *normative regularization*, i.e., statistical regularization that is informed by normative coding principles, such as the idea that sensory systems have evolved to efficiently process natural stimuli. Specifically, we used this strategy to incorporate EC as a regularizer and developed a hybrid model that combines SI-based neural prediction and EC in a single model. The two model branches are linked by shared convolutional filters (Fig 1b).

The *SI branch* approximates the response functions of recorded neurons to a visual dense noise (see below), and was implemented using a convolutional neural network (CNN) (Fig 2a). Here, we used an L2 regularization on the convolutional layers to encourage smooth filters [53] and an L1 regularization on the fully connected (FC) layer for sparse readouts ([19]; for details, see Methods).

The *EC branch* was trained to efficiently reconstruct input stimuli (i.e., natural scenes) from a constrained latent representation. For this branch, we used a convolutional autoencoder network that we published before (for details, see [39] and Methods). Also in the EC branch, we enforced smooth filters by using L2 regularization. In addition, we limited the bandwidth by adding Gaussian noise and imposing L1 regularization on the hidden activations. The latter regularization also encourages sparse representations.

In the *hybrid model*, we implemented interactions between the two branches by shared filters (symbolized by red circle in Fig 1b). Both branches were trained in parallel, with a weighted sum of their respective losses (*L*_*SI*_ and *L*_*EC*_) used as optimization objective. By changing the weighting of the two losses, we were able to control the relative contribution of two branches on shaping the shared filters, and test our hypothesis to which degree efficient representations of natural scenes improve neural predictions (Fig 2a and 2b). Specifically, weight *w* was used to define the hybrid model’s loss function as *L*_*Hybrid*_ = *w* ⋅ *L*_*SI*_ + (1 − *w*) ⋅ *L*_*EC*_ (Methods). For *w* = 1, the EC branch had no influence on the shared filters and, hence, the hybrid model behaved like the pure SI model. Conversely, for *w* = 0, the SI branch had no influence on the shared filters and, hence, the hybrid model behaved like the pure EC model. Thus, the smaller the weight, the more the EC branch contributed to shaping the filters.

To evaluate the influence of stimulus statistics on neural response predictions, we fed not only natural stimuli to the EC branch, but also phase-scrambled natural stimuli as well as noise. We refer to these models as *hybrid-natural*, *hybrid-pha-scr* and *hybrid-noise* (Fig 2c). Moreover, to examine whether the performance improvements could be attributed to simple low-pass filtering, we trained SI networks using spatial convolutional filters composed of different numbers of basis functions derived from principle component analysis (PCA) on natural images (Fig 2d), or the discrete cosine transform (DCT). These models are referred to as *SI-PCA* and *SI-DCT* networks.

To train the SI branch of our hybrid framework, we recorded somatic Ca^2+^ responses from populations of cells in the ganglion cell layer (GCL) of the *ex-vivo* mouse retina to 9-minute long noise stimuli using two-photon imaging (Fig 3a; Methods; [44, 47]). The GCL contains the RGCs, which represent the retina’s output neurons and form in the mouse about 40 parallel feature channels to higher visual brain areas (reviewed in [23]). RGCs gain their specific response properties by integrating upstream input from distinct sets of bipolar cells and amacrine cells. Note that the GCL also contains some “displaced” amacrine cells (dACs; [47, 59]). If not indicated otherwise, we did not distinguish between these two GCL cell classes in our datasets. The noise stimulus contained two chromatic components (UV, green) matching the spectral sensitivities of mouse photoreceptors [60]. We used the data of n = 96 GCL cells that passed our quality criteria (Methods) to fit a pure SI model with factorized spatial and temporal convolutional filters, whose predictive performance served as our baseline (Fig 3b left).

### Neural system identification benefits from natural scene statistics

First, we measured the predictive performance of the *hybrid-natural* model on the validation data (for hyperparameter tuning) by systematically varying the relative impact of the two branches, i.e., changing the weight *w*. We found that the performance steadily increased with increasing EC influence (i.e., decreasing *w*) up to an optimum (peaking at *w* = 0.2; Fig 3c, red), after which the SI had too little influence on the shared filters and the performance dropped.

Next, we replaced the natural input to the EC pathway by phase-scrambled scenes (*hybrid-pha-scr*) and white noise across space and chromatic channels (*hybrid-noise*). Like for the *hybrid-natural* model, the performance of the two control models also increased with increasing EC influence up to a certain point, peaking at *w* = 0.3 and *w* = 0.4 for *hybrid-pha-scr* and *hybrid-noise*, respectively (Fig 3c). This indicates that when incorporating EC, all hybrid model versions showed some improvement up to certain *w* values, before performance sharply declined.

To test to what extent simple low-pass filtering contributes to the performance improvement observed for the *hybrid-natural* model, we quantified the performance of two additional SI models, one with PCA and the other one with DCT bases. By varying the number of bases used, we found a maximum in predictive performance at 16 and 4 bases for *SI-PCA* and *SI-DCT* (zig-zag ordering), respectively (S1(b) Fig).

Finally, to compare the performance on the test data across models, we picked for each model the *w* or number of bases with the best predictive performance for the validation data. We found that the hybrid model with natural inputs to the EC branch attained the best performance among all tested models (Fig 3d and 3e). The *hybrid-natural* model’s superior performance compared to the *hybrid-pha-scr* model suggests that the benefit of learning natural scene statistics extends beyond second-order statistics such as the 1/*f* power spectrum of natural images. Nevertheless, the *hybrid-pha-scr* model performed better than the *hybrid-noise* version, pointing at a general benefit of learning second-order statistics in the EC branch. Moreover, the *hybrid-natural* model was consistently better than low-pass filtering control models (*SI-PCA* and *SI-DCT*), suggesting that simple low-pass filtering does not fully explain the benefits of sharing kernels with the EC branch trained to efficiently represent natural stimuli.

Together, our results suggest that normative network regularization—in particular, based on natural statistics—can improve the performance of neural SI models on predicting responses to noise.

### Hybrid models with natural inputs learn the most “biologically-plausible” filters

To confirm that our hybrid models capture the properties of the recorded cells, we estimated their RFs (Fig 3b and S1(f) Fig; Methods). Indeed, we found that the models learned antagonistic center-surround RFs with biphasic temporal kernels, reminiscent of RGC RFs found in other studies [2, 47]. To get insights to which degree our models resembled biological vision systems, we next investigated the internal representations by analyzing the filters of the models’ subunits [18, 61]. To this end, we compared the shared spatial convolutional filters between our tested models. As neurons in the retina and further upstream in the early visual system often feature smooth, Gaussian or DoG shaped RFs (e.g., [47, 62, 63]), we refer in the following to models with such shared filters as more “biological plausible” than those with other filter organizations.

Interestingly, while the learned neuronal RFs were quite consistent between models (cf. Fig 3b), their shared spatial filters differed considerably (Fig 3f and 3h). When using natural images in the EC branch (*hybrid-natural*), filters indeed became smoother and more Gaussian-shaped, which may be due to the regularization by the EC branch on the SI branch and may have contributed to the performance improvement of predicting responses. This effect persisted though reduced when phase-scrambled images were used (*hybrid-pha-scr*). Moreover, for smaller *w* values (i.e., stronger EC influence), Gaussian-shaped filters became more frequent in the *hybrid-natural* but not in the *hybrid-noise* model (Fig 3f, upper vs. lower row). For the SI models with PCA or DCT basis, we found all filters to be smooth as they profited from low-pass filtering of the respective transformation. However, compared to the *hybrid-natural* model, their filters were less frequently Gaussian-shaped (Fig 3h).

To quantify these findings, we fit 2D Gaussian functions to the filters and measured the goodness of the fit via the coefficient of determination (R-squared; Methods). Notably, for all three hybrid models, the *w* with the best Gaussian fit was the same *w* that also resulted in the best response predictive performance (*w* = 0.2, *w* = 0.3, and *w* = 0.4 for *hybrid-natural*, *hybrid-pha-scr*, and *hybrid-noise*, respectively; Fig 3g). The filters of the *hybrid-natural* model resembled smooth 2D Gaussians more than for any other model (Fig 3i), including *SI-PCA* and *SI-DCT*. The difference of fit quality between *hybrid-natural* vs. *hybrid-pha-scr* and *hybrid-pha-scr* vs. *hybrid-noise* may be related to higher-order statistics and second-order statistics of natural scenes, respectively.

Taken together, our comparisons of the hidden spatial representations suggest that natural scene statistics promote latent feature representations akin to transformations in the early visual system.

### Efficient coding increases the data efficiency of system identification

**Fig 4:**
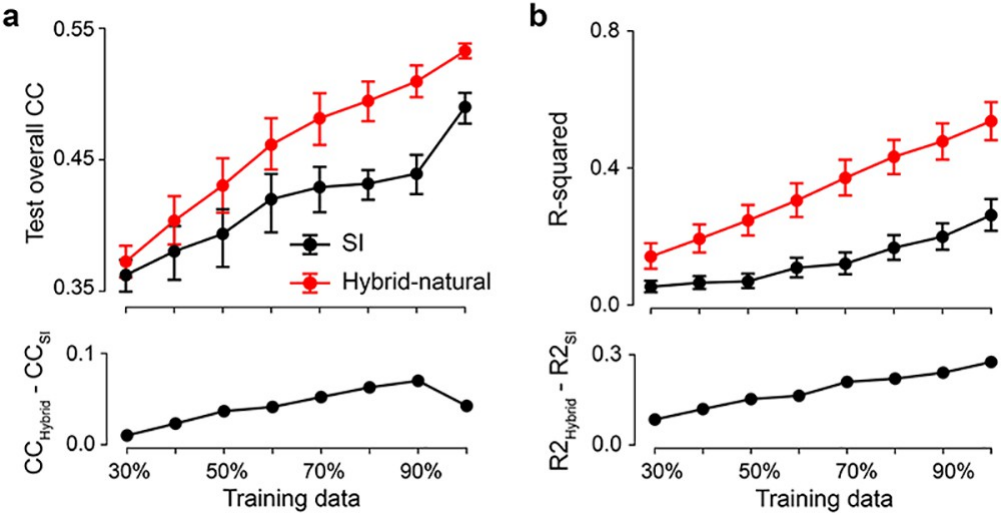
*Hybrid-natural* models have better data efficiency for neural prediction. **a.** Mean model performance (top) based on test data for SI and *hybrid-natural* (*w* = 0.2; *n* = 10 random seeds) with different training data sizes and mean difference between SI and *hybrid-natural* (bottom). **b.** Mean R-squared (top) of fitting a 2D Gaussian to spatial filters for green stimulus channel for SI and *hybrid-natural* (*w* = 0.2; *n* = 10 random seeds) with different training data sizes, and the mean difference between R-squared for SI and *hybrid-natural* (bottom). Error bars represent 2.5 and 97.5 percentiles with bootstrapping.

Next, we asked if the observed performance increase in the *hybrid-natural* vs. the baseline SI model was sensitive to the amount of training data, both with respect to their response predictions (Fig 4a) and their learned spatial filters (Fig 4b). To this end, we trained the SI and the *hybrid-natural* model (*w* = 0.2) with different amounts of data, ranging from 30% to 100%.

Not unexpectedly, when more training data was used, predictive performance increased for both models (Fig 4a top). However, we also found that the performance of the *hybrid-natural* model was consistently higher than that of the SI model, with the difference becoming significant for ≥ 60% and peaking at around 90% training data (Fig 4a bottom). Additionally, for both models the spatial filters became increasingly more Gaussian-like with more data (Fig 4b). We also observed that the performance difference dropped for large dataset sizes—which, we expect, is asymptotically near zero in the regime of infinite data.

Together, these results suggest that a hybrid model predicting responses to noise, but with access to natural statistics requires significantly less training data than the baseline SI model.

### Hybrid models for testing temporal coding strategies

It has been suggested that early stages of visual processing, rather than encoding a past stimulus (past encoding), aim at predicting future stimuli in their temporal stream of inputs [64]. Such a future prediction strategy is thought to extract information that can be used for guiding future behavior [65]. Therefore, we next tested if sharing spatio-temporal (i.e., 3D) filters can further improve the predictive performance of the hybrid model. We implemented both strategies—past encoding and future prediction—in the EC branch, and compared their influence on the SI task [66].

We modified the 2D SI model to use spatio-temporal (instead of factorized spatial and temporal) convolutional filters to predict neural responses for 8-frame noise movies (3D SI model; S2(a) Fig). Likewise, we employed spatio-temporal convolutional filters for the EC branch. As before, the two branches of the resulting hybrid model were trained in parallel, but now sharing spatio-temporal filters. In the past encoding case, the EC branch was trained to reconstruct the 7^th^ frame (at *t* − 1) of a continuous 8-frame natural movie clip based on frames at *t* − 7 to *t* (*hybrid-natural-past*; S2(b) and S2(c) Fig). In the future prediction case, the EC branch was trained to predict the 8^th^ unseen frame based on the first 7 frames (*t* − 7 to *t* − 1) of the clip (*hybrid-natural-future*; S2(d) Fig left).

**Fig 5:**
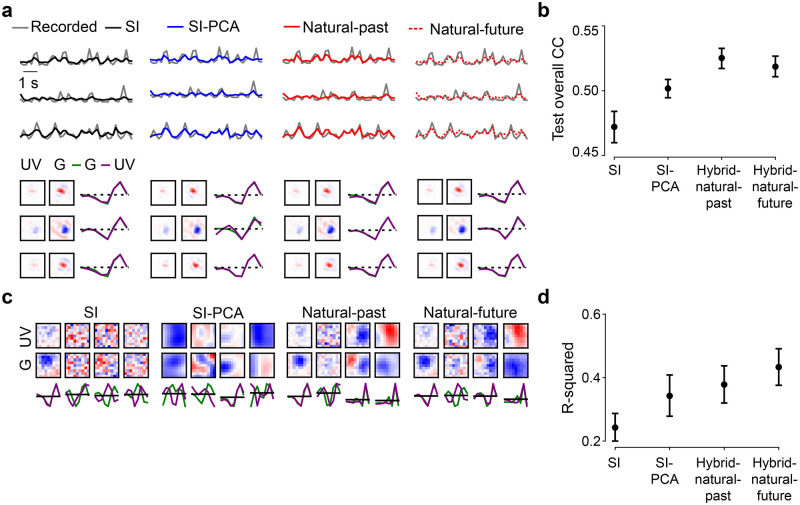
Past encoding or future prediction strategies using 3D shared filters perform equally well. **a.** Top row: Responses of three exemplary GCL cells to 5-Hz noise stimulus (gray) and predictions of best performing models on test data (black, SI; blue, SI with PCA filters; red solid, hybrid for encoding the past; red dotted, hybrid for predicting the future). Bottom row: Respective learned RFs of the three cells (visualized by SVD). **b.** Mean model performance based on test data for SI, SI-PCA (128 bases), *hybrid-natural-past*, and *hybrid-natural-future* (both *w* = 0.4; *n* = 10 random seeds; *p* < 0.0001 for SI vs. *hybrid-natural-past*, *p* = 0.0005 for SI-PCA vs. *hybrid-natural-past*, *p* = 0.2563 for *hybrid-natural-past* vs. *hybrid-natural-future*, two-sided permutation test, *n* = 10, 000 repeats). **c.** Representative shared spatial and temporal filters of 3D models (n = 1 random seed, visualized by SVD; temporal kernels for UV and green stimulus channels indicated by purple and green, respectively). **d.** Mean R-squared of fitting a 2D Gaussian to shared spatial filters (for green stimulus channel; *n* = 10 random seeds per model; *p* = 0.0003 for SI vs. *hybrid-natural-past*, *p* = 0.4356 for *SI-PCA* vs. *hybrid-natural-past*, *p* = 0.1895 for *hybrid-natural-past* vs. *hybrid-natural-future*, two-sided permutation test, n = 10,000 repeats). Error bars in (b),(d) represent 2.5 and 97.5 percentiles with bootstrapping.

Like for the 2D models, we varied *w* or the number of bases and then selected the best model for each condition (3D SI, *hybrid-natural-past*, *hybrid-natural-future*, and 3D *SI-PCA*) based on validation performance. We next quantitatively compared the different models using the test data (Fig 5a and 5b; S3c Fig). We found that the 3D *SI-PCA* model outperformed the 3D SI model, presumably because the former profited from the low-pass filtering of the PCA transformation. Importantly, both hybrid models displayed a better performance than the 3D *SI-PCA* model. While the *hybrid-natural-past* model performed slightly better than its *hybrid-natural-future* counterpart, this difference was not statistically significant. In summary, both the past encoding and future prediction strategy in the EC branch turned out to be equally beneficial for predicting the responses to noise stimuli and, as before, the benefit extended beyond low-pass filtering effects. However, no performance increase was achieved with respect to the 2D *hybrid-natural* model (Fig 5b vs. Fig 3d).

We also analyzed the shared spatio-temporal filters using the same metric as for the 2D case, which assesses the similarity between spatial filters (after performing a low-rank decomposition of 3D shared filters into spatial and temporal components; see Methods) and smooth 2D Gaussians (Fig 5c and 5d). Again, we found higher R-squared values for the hybrid models and the 3D *SI-PCA* model compared to the baseline SI case. Note that here, the 3D *SI-PCA* model did not significantly differ from the two hybrid models, possibly due to a large number of bases (*n* = 128 vs. *n* = 16 in the 2D case).

Next, we asked if the fact that we did not see a significant advantage of 3D over 2D could be because the slow (5 Hz) noise stimulus did not sufficiently drive GCL cell responses. Therefore, we recorded a dataset (*n* = 64 cells), in which we presented a 30-Hz dense noise stimulus and used it with the 3D hybrid models. Like for 5-Hz noise, the *hybrid-natural-past* and *hybrid-natural-future* models performed better than the 3D SI model, both on response prediction and with higher R-squared values for the learned filters (S4 Fig). But again, the 3D hybrid models performed only equally well compared to their 2D counterparts.

In summary, the *hybrid-natural* models achieved a higher performance for different noise stimuli (5-Hz vs. 30-Hz) and different shared filter organizations (2D vs. 3D) than all other tested models. Therefore, it is likely that their superior predictive performance for neuronal responses and their more biologically plausible filters resulted from the EC branch having access to natural statistics.

### Direction-selective neurons benefit more than others from hybrid models

**Fig 6:**
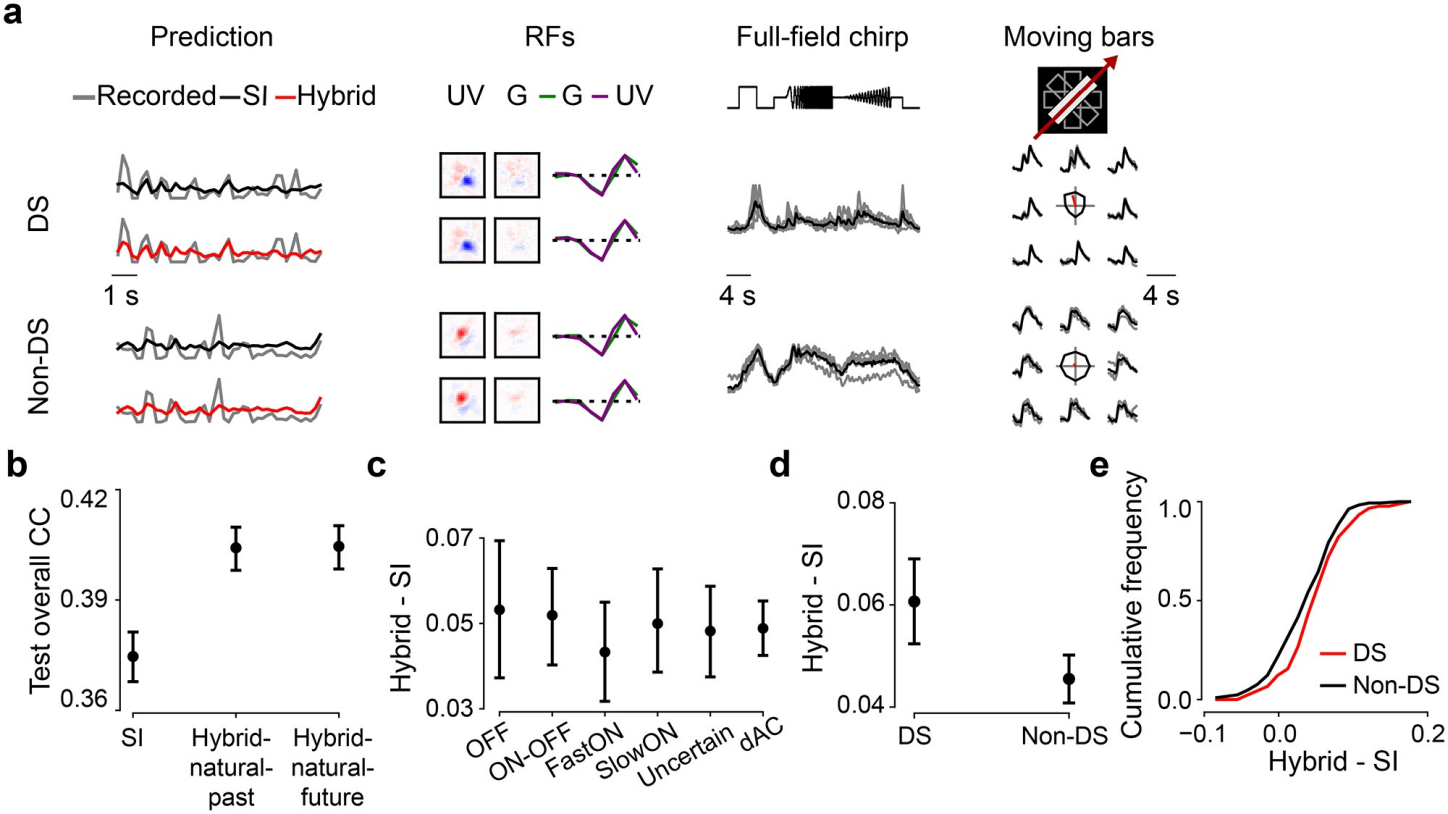
Direction-selective (DS) neurons benefit more from hybrid models. **a.** Recorded (gray) and predicted (black, SI; red, *hybrid-natural-past*; response amplitude scaled with a constant 1.5 for better visualization) responses to noise, RFs, as well as full-field chirp responses and moving bar responses (gray, single trials; black, means) of representative DS and non-DS cells. Note that the RFs were dominated by UV stimulus channel because cells were recorded in ventral retina (see Methods). **b.** Mean model performance based on test data for SI, *hybrid-natural-past* and *hybrid-natural-future* (both *w* = 0.7; *n* = 10 random seeds per model; trained with responses of *n* = 427 GCL cells to 5-Hz noise stimulus; *p* < 0.0001 for SI vs. *hybrid-natural-past*, *p* = 0.9307 for *hybrid-natural-past* vs. *hybrid-natural-future*; two-sided permutation test, *n* = 10, 000 repeats). Note that compared to Fig 5b, these models had a lower predictive performance, as we used a different dataset, with 30% of data for training. **c.** Difference in mean performance between *hybrid-natural-past* and SI based on test data for 6 broad functional groups of GCL cells (35 OFF, 59 ON-OFF, 49 fast-ON, 38 slow-ON, and 64 uncertain RGCs, as well as 145 dACs; see Methods and Results; *n* = 10 random seeds per model). **d.** Like (b) but for *n* = 90 DS and *n* = 300 non-DS cells. **e.** Cumulative histogram of difference in mean prediction between *hybrid-natural-past* (*w* = 0.7) and SI on test data for DS (red) and non-DS cells (black), at one particular seed. Error bars in (b)–(d) represent 2.5 and 97.5 percentiles with bootstrapping.

The retina encodes the visual scene in a number of features that are represented by more than 40 different types of RGC whose outputs are relayed in parallel to higher visual centers in the brain [47, 67–70]. Thus, we next asked, if access to natural statistics allowed our hybrid models to predict some cell types better than others (Fig 6). Earlier, it has been shown that motion-relevant properties emerge in the efficient coding framework for both past encoding and future prediction approaches [66]. Therefore, we employed our 3D hybrid models (cf. Fig 5) and focused on direction-selective (DS) cells [47, 71].

For this analysis, we used a set of *n* = 427 GCL neurons, whose responses were recorded not only to the 5-Hz noise stimulus (for training the models) but also to full-field chirp and moving bar stimuli. The latter two stimuli (Fig 6a) enabled us to identify the functional type of each recorded GCL neuron [47] using a cell type classifier (see Methods; S5 Fig).

We observed that for 100% of the data, SI and hybrid model performed similarly well. For the analysis of cell type-specific performance, we therefore chose a dataset size (30% of total recording time) for which the predictive performance difference between the two models was particularly pronounced. As expected, we found that both hybrid networks (*hybrid-natural-past* and *hybrid-natural-future*) performed significantly better than the SI model, with no significant difference between the two hybrid models (cf. Fig 5b, S4(b) Fig).

First, we evaluated if any of the broader functional groups of GCL cells profited more from natural statistics than others. For this, we sorted the cells into 6 groups based on their response polarity (ON vs. OFF) and transience, and based on whether they were RGCs or dACs (for group sizes, see Fig 6 legend). For all 6 groups, the hybrid models showed a better predictive performance than the SI model (Fig 6b). However, no significant differences were observed between any pair of groups (*p* > 0.05 for all pair-wise comparisons, two-sided permutation test, n = 10,000 repeats; Fig 6c) and the two hybrid models (*p* > 0.05 for all pair-wise comparisons; S6(a) Fig).

Next, we grouped the cells into DS (*p* < 0.05, direction tuning using a permutation test; *n* = 90) and non-DS cells (*n* = 300) based on their moving bar responses (Fig 6a right). Note that *n* = 37 neurons were excluded as they did not pass the quality test for chirp and moving-bar responses (Methods). We found that the predictive performance for DS cells was significantly higher than that of the non-DS cells for both *hybrid-natural-past* (Fig 6d and 6e; *p* = 0.0027) and *hybrid-natural-future* (S6(b) and S6(c) Fig; *p* = 0.0042). To test whether this performance difference was merely due to different signal-to-noise ratios in DS vs. non-DS cells, we compared their response quality indices (*QI*; Methods). While DS cells had significantly higher *QI* values for moving-bar responses (*QI*_*bar*_) than non-DS cells, we did not find any significant difference between the two groups with respect to their noise (*QI*_*noise*_) or chirp responses (*QI*_*chirp*_; S6(e), S6(f) and S6(g) Fig). These results suggest that DS cells benefit more from the EC branch of the hybrid models than non-DS cells, partially consistent with earlier findings ([66]; see also Discussion).

In summary, efficient coding of natural statistics served as a beneficial normative regularization for predicting responses to noise stimuli of all types of mouse GCL cells and in particular DS cells, suggesting the potential role of motion statistics in the natural environment on shaping neuronal response properties.

### Hybrid models for predicting retinal responses to natural movies

Natural stimuli are thought to drive more diverse neural responses compared to artificial stimuli, such as dense noise or drifting bars [72]. As a result, more complex feature transformations are expected to be required for determining the respective stimulus-response functions ([18, 73], but also see [74]). Therefore, we tested if predicting neural responses to natural movies would also profit from our hybrid model.

**Fig 7:**
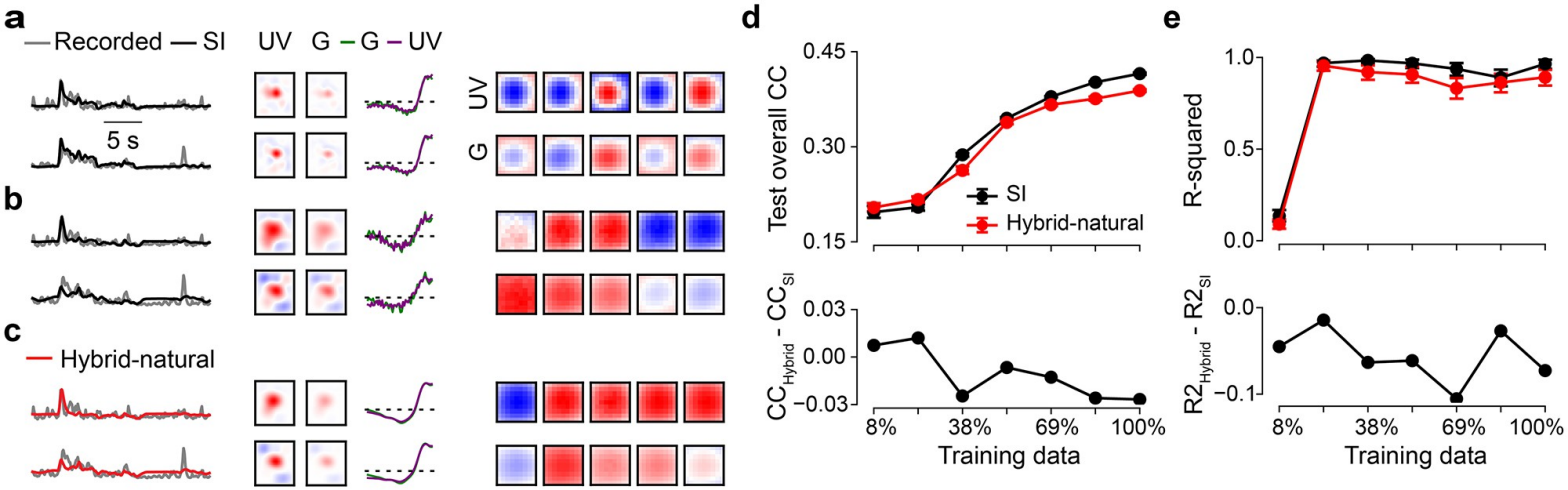
Predicting neural responses to natural movies does not benefit from efficient coding. **a.** Recorded (gray) and predicted (black, SI; red, *hybrid-natural*) responses to natural movie, RFs, as well as exemplary spatial filters for the SI model trained by full training data. **b.** Same as (a), but for the SI model trained by 23% of training data. **c.** Same as (a), but for the *hybrid-natural* model trained by 23% of training data. **d.** Predictive performance (top) based on test data for SI and *hybrid-natural* (*w* = 0.4; *n* = 10 random seeds) with different training data sizes, and the difference between SI and *hybrid-natural* (bottom). **e.** R-squared (top) of fitting a 2D Gaussian to spatial filters for UV stimulus channel for SI and *hybrid-natural* (*w* = 0.4; *n* = 10 random seeds) with different training data sizes, and the difference between SI and *hybrid-natural* (bottom). Error bars in (d),(e) represent 2.5 and 97.5 percentiles with bootstrapping.

To this end, we used the neural activity of *n* = 86 ventral GCL neurons that were presented with 30-Hz natural movies ([39]; Fig 7a left) to train a stand-alone SI model with factorized spatial and temporal filters. Surprisingly, the SI model learned center-surround RFs with biphasic temporal components as well as smooth 2D Gaussian spatial filters with high R-squared values (mean R-squared = 0.96; full training data of 433 s; Figs 7a, 3f, 3h and 4b).

Therefore, we next tested if there was a performance difference between the SI and hybrid models for less training data (Fig 7b and 7c). Here, we decided to use approx. a quarter of the data (i.e., 23%, or 15 of 65 mini-batches). As with the models using neural responses to noise, we tuned the hyperparameters based on validation data (*w* = 0.4 for *hybrid-natural*) and fixed them. We then evaluated the performance of the two models after being trained with different amounts of data, ranging from 8% to 100%. As expected, predictive performance of both models increased with available data. However, compared to the models trained with noise responses, the *hybrid-natural* model had similar predictive performance with the SI model, with only a marginal improvement for small amounts of data (<25%) (Fig 7d; cf. Fig 4a; see Discussion).

Additionally, the two models had similar R-squared values across different data sizes (*hybrid-natural* model with slightly lower values; Fig 7e), indicating that they learned filters that resembled 2D Gaussians comparably well. We also observed that both models featured filters with high R-squared values starting from 23% of the data (cf. Fig 4b).

Together, these results suggest that normative network regularization, as implemented in our hybrid models, offers no additional benefit for predicting responses to natural movies.

## Discussion

In this study, we asked if access to natural scene statistics can help predicting neural responses. To address this question, we combined system identification (SI, [3]) and efficient encoding (EC, [25]) methods into a normatively regularized (hybrid) modeling framework. Specifically, we used models that efficiently represent natural scenes recorded in the mouse’ habitat to regularize models that predict retinal responses to visual stimuli. We analyzed such hybrid models with shared spatial filters, and found that natural images as input to the EC branch indeed improved the performance in predicting retinal responses to noise stimuli and allowed the model to generate filters that resembled RFs found in the early visual system. These improvements extend beyond those gained by simple low-pass filtering or using second-order statistics of the natural scenes. Our hybrid models with shared spatio-temporal filters performed similarly well as those with shared spatial filters, independently of whether they used a past encoding or a future prediction strategy. Notably, predictions of DS cell responses to noise stimuli improved the most in the hybrid models with natural input to the EC branch. Interestingly, in predicting neural responses to natural movies, both hybrid and SI models performed similarly well. In summary, our results suggest that sourcing information about an animal’s environment—e.g., through hybrid SI-EC models—can help building more predictive and biologically-plausible models of neuronal networks—at least when predicting neural responses to artificial stimuli and/or for limited amounts of data. More generally, our findings lend support to the idea that knowledge of natural statistics is already encoded in sensory circuits.

### Hybrid models improve data efficiency

When predicting responses to noise, the difference in predictive performance between the hybrid and the baseline SI model was significant and it depended on the amount of available data, indicating that our hybrid modeling approach increased data efficiency. The data efficiency also depended on the input to the SI branch in the hybrid models: For natural stimuli, the performance gain was marginal and restricted to the case when data was strongly limited. Therefore, we expected our hybrid models to improve SI mainly when only little neural data in response to artificial stimuli is available. It is possible that for those more challenging problems at downstream visual areas, where neural response functions and, hence, the neural prediction tasks, become more complex [75], the data efficiency of a hybrid approach and the improvement from natural scene statistics may be higher.

### Biological plausibility and temporal coding principles in hybrid models

Regarding the spatial filters, for most learned models the degree of similarity to Gaussian RFs was positively correlated with their predictive performance (with the exception of the *SI-DCT* models)—whether there is a causal link remains unclear (see below). Note that we used the filters’ similarity to 2D Gaussian functions as a proxy for biological plausibility, following the assumption that for RFs in the retina and at early downstream stages of the visual system, a smooth, Gaussian-like structure is often a suitable approximation (e.g. [47, 62, 63]). Accordingly, the fitted Gaussian shapes had diameters of 4–9 pixels, equivalent to 3.3°-7.4° of visual angle and, hence, in the range of RF center sizes of mouse RGCs (3°-13°; [76–78]). However, it has been reported that RGCs, for instance, can also feature multiple sensitivity peaks and irregular non-Gaussian shapes in their RF (e.g., [79]). Therefore, our proxy of biological plausibility may underestimate the complexity of retinal representations and for future studies, it would be important to use additional filter properties, such as locality and smoothness, as metrics. Moreover, a deep, systematic understanding of artificial and neuronal networks and their hidden representations likely calls for other methods besides filter inspection, e.g., the evaluation of temporal curvature ([80, 81]; discussed in [82]). As the natural environment is not static, we also created hybrid models that acknowledged the time domain by sharing spatio-temporal filters. Surprisingly, both variants—past encoding and future prediction—behaved quite similarly. Note that our future prediction approach is not the same as “predictive coding“, which removes redundancy in uniform or correlated inputs by encoding the difference between the actual input and the internal expectation [24, 83–85]. However, in the stand-alone EC models (that is, only the EC branch), the temporal components of the filters learned by the future prediction were much more diverse than those of past encoding (S2(c) and S2(d) Fig right). Interestingly, the differences between temporal filters of these stand-alone EC models decreased with the incorporation of the neural prediction task in the hybrid models.

The filter diversity in our 3D hybrid models is reminiscent of earlier findings by Chalk and colleagues [66], who reported the emergence of filters sensitive to motion direction and motion speed in their past encoding and future prediction EC models, respectively. However, in contrast to their results, we did not see a difference between our *hybrid-past* and *hybrid-future* models with respect to motion-sensitive filters: Both of them performed better in predicting responses of DS vs. non-DS cells. Further work is needed to understand that partial (mis)match between our work and that by Chalk et al., and why specifically DS cells profited from both our 3D hybrid models. It is possible that the better performance for DS cells is related to the fact that the natural movies we used for training the EC branch are dominated by global motion [39]. In other words, our EC model may be prone to produce filters that detect spatio-temporal structures inherent in the training input.

### Hybrid models of retinal signal processing

Only for very limited data, our hybrid models displayed a marginal improvement when predicting neural activity to the natural movie stimulus vs. the noise stimulus. This was surprising, as we expected that the EC branch supports the learning of complex feature representations driven by natural stimuli [18, 73]. That the stand-alone SI model trained with natural movie responses easily learned smooth Gaussian filters may have limited the benefits from the hybrid model. In turn, this may indicate that, indeed, predictive model performance correlated with biological filter plausibility. To further explore the interaction between learned filters and predictive performance, it may be instructive to test a greater variety of stimuli and record responses to them from the same neurons. Such data may also be useful for characterizing model generalization (domain transfer, see e.g., [73, 86]) by using responses to natural stimuli as unseen test data with a hybrid model trained with cell responses to noise stimuli. Here, one would need to take into account that RGCs may adapt to different stimuli (such as noise vs. natural movies) by changing their RF properties [84].

Many studies have applied EC principles to natural images with different regularization strategies, such as adding noise (to the input, hidden activation or output), forcing sparsity (of weights, hidden activation or responses), and encouraging smoothness and spatial locality of weights. These coding principles produced diverse feature representations, including DoG and Gabor filters [38, 39, 87–89]. For example, Doi et al. [89] found that the response sparsity and the spatial locality of filter weights induced oriented and center-surround structures, respectively. In our previous study [39], we tested a convolutional autoencoder using a smoothness constraint (L2 regularization) on the convolutional/deconvolutional filters, and a sparsity constraint (L1 regularization) as well as Gaussian noise on the encoder output. We found that this model produced center-surround filters when trained with natural images. Similarly, Ocko et al. [38] trained an autoencoder model with pink noise and obtained DoG filters. Our study shows that *hybrid-natural* had a better predictive performance and biological plausibility than *hybrid-pha-scr*. In turn, *hybrid-pha-scr* outperformed *hybrid-noise*. This suggests that both the regularization and the statistical properties of the model input we used contributed to the emergence of center-surround features.

Generally, the effect of normative network regularization depends on many factors, including—in our hybrid models—neural prediction tasks (e.g., predicting responses to noise vs. natural movies), normative principles (e.g., encoding the past vs. predicting the future), stimulus input of the EC branch (e.g., noise vs. natural scenes), and shared components between two branches (e.g., filter weights vs. network features). Any of them may influence the model performance or the learned filter representations. A factor that we did not vary much was nonlinearity of the model. For example, that our hybrid approach did not improve the prediction of responses to natural movies, which are highly non-linear and complex [18, 73], may be due to limited expressive power of the EC network and shared units.

For our current analysis, we used broad group assignments (e.g., Fast ON RGCs), which include several functional types of RGC (e.g., ON-step, ON-transient, ON-high-frequency etc; [47]) or dACs, but did not detect any differences in performance gain except for the DS neurons. Still, it is possible that distinct types of RGC profit more than others from the EC branch of our hybrid models. For example, the so-called W3 RGCs, for which the best stimulus found so far is a small dark moving spot [90], may not be “designed” to efficiently represent natural stimuli but rather to extract survival-relevant features (i.e., detecting aerial predators). Here, we could build models with different normative regularization or tasks (i.e., detecting predators in images of the sky) and would expect that this RGC type profits little from efficiently encoding natural statistics in the hybrid model. In this way, we may be able to discover the computational functions of specific cell types. Studying coding strategies across RGC types could contribute an important biological perspective to the perennial debate between efficient coding [91] and feature detection [67] proponents.

### Normative network regularization as a framework for studying neural coding

In this study, we regularized the filters of a SI model with a normative EC model to predict visually-evoked responses of cells in the retina, which could be seen as a multitask learning model [92]. This approach is not limited to a combination of EC and SI, for example, Yamins et al. [11] used a model trained on an image categorization task for neural prediction in a sequential way. Some forms of normative regularization have also been discussed and/or applied in earlier work. For example, Deneve and Chalk [93] discussed the relations between SI (encoding) models and EC, and argued that the latter may promote shifting the focus in SI from the single-cell to the population level. The integration of stimulus-oriented approaches (such as EC) for discriminative tasks (such as object recognition) was proposed by Turner et al. [15]. Later, Teti et al. [94] employed sparse coding with lateral inhibition in simulations of neuronal activation in visual cortex. More recently, Młynarski et al. [42] presented a probabilistic framework combining normative priors with statistical inference and demonstrated the usefulness of this approach for the analysis of diverse neuroscientific datasets. However, their work was rather conceptual, with the datasets they used being either simulated or low-dimensional. Notably, they tested their framework on pre-fit retinal RFs, but not directly on actual RGC stimulus-response data. Compared to their framework, our method does not require marginalization across all parameter space to estimate optimality and could be applied to more general or complex inference problems. Hence, our work not only provides further evidence to the feasibility of combining coding principles for identification of neural response properties on high-dimensional data, it also demonstrates the benefits of leveraging natural scene statistics for neural prediction. However, compared to the framework by Młynarski et al., with our approach it is more difficult to conduct rigorous statistical tests of normative theory.

We expect that our hybrid modeling strategy for prediction of responses to noise stimuli may also work for different processing stages along the early visual pathway (and potentially other modalities, e.g., sound). This said, however, one needs to keep in mind that different stages along the visual pathway have different tasks and constraints, and, thus, likely incorporate different efficient coding principles: For instance, the retinal hardware is space-limited and has to encode visual features in view of a bottleneck with limited bandwidth (optic nerve), whereas the primary visual cortex has comparably abundant resources which might serve for accurate probability estimation for behavioral tasks, such as novelty detection (discussed in [24, 95]). It is also worth to note that different visual processing stages (such as primary visual cortex vs. higher visual areas, or adaptation of visual coding to different behavioral states) may benefit from the hybrid modeling to a different degree, as efficient coding approaches learn filters that may be more relevant to stimulus-related features, but not high-level behavior goals (see discussion in [15]). Additionally, it would be interesting to compare our hybrid models with SI models regularized with other behavioral tasks such as object recognition (e.g., [11]) or predator detection (see above) for neural predictions along the ventral visual stream.

While this study focused on normative regularization for neural prediction task, it would be also interesting to infer EC principles from stimulus-response data. With our framework, a possible starting point could be to compare a normative criterion, such as image reconstruction fidelity, between the hybrid model and a stand-alone EC model. Such analysis could be extended by either evaluating the difference for coding principles with the use of the same stimulus-response data, or testing a normative criterion using different experimental datasets.

There is a long tradition of using SI models (reviewed in [3]) in predicting the responses of neurons to a great variety of stimuli (e.g., [2, 4, 18, 19, 96, 97]). Our results demonstrate how the EC hypothesis can be successfully leveraged as normative regularization for the identification of neural response properties when assessed through noise stimuli. Additionally, predicting the response to naturalistic stimuli may be more beneficial for learning biologically-plausible filters. More generally, the hybrid framework offers an opportunity to test different coding principles and unsupervised learning objectives with regards to experimental data for understanding neuronal processing.

## Supporting information

S1 Fig Training of 2D models. **a.** The noise stimulus (9 minutes in total) containing training and validation data (1 repeat) and test data (6 repeats). **b.** Model performance (mean) based on validation data for SI-PCA and SI-DCT with different numbers of basis. SI-PCA and SI-DCT yielded best performance when using 16 and 4 bases, respectively (each model for n = 10 random seeds; error bars represent 2.5 and 97.5 percentiles with bootstrapping). **c.** Training loss as a function of training epochs for the hybrid model (Input_EC_, natural scenes) with different weights (*w*), indicated by color (right). **d.** Model performance based on validation data (with linear correlation coefficient as metric) during the *hybrid-natural* model training with different weights (colors as in (c)). As weight decreased from 1 to 0.2, more training epochs were needed to reach the best performance. The hybrid model performed best for *w* = 0.2. Note that the hybrid model showed a slower change in correlation coefficient (CC) around the peak at *w* = 0.2 (compared to *w* = 1), demonstrating the regularization effects of the EC branch on the hybrid model. **e.** Scatter plots for model predictions based on test data at a particular seed (each dot representing one neuron). Hybrid with natural scenes as input_EC_ (*w* = 0.2) vs. SI, SI with PCA basis (16 bases), SI with DCT basis (4 bases), *hybrid-pha-scr* (*w* = 0.3) and *hybrid-noise* (*w* = 0.4). **f.** Upper: Three representative GCL cell responses (gray traces) to noise stimulus together with predictions of the best performing models on test data (black, SI; blue, SI with PCA basis; cyan, SI with DCT basis; red, hybrid w/ natural scenes as input in EC path; brown, hybrid w/ phase-scrambled scenes as input in EC path; magenta, hybrid w/ noise as input in EC path). Lower: Learned spatio-temporal RFs of the example cells, visualized by SVD. Same random seed as in (e).
(TIF)

S2 Fig Three-dimensional hybrid networks embedding natural movies. **a,b.** Illustration of SI network (a) with 3D spatio-temporal convolutional filter, and EC network (b), reconstructing the 7^th^ frame (at *t* − 1) based on 8 continuous frames (*t* − 7 to *t*; encoding the past, c). Combined as a hybrid network, the two branches were trained in parallel with shared 3D filters (all spatio-temporal filters were shared; Input_EC_, 8-frame UV-green movie clip; Output_EC_, reconstruction of the 7^th^ frame of Input_EC_). **c.** Example for input/output of the EC model for encoding the past (left; also see b) and exemplary spatio-temporal convolutional filters when using natural movies as input to train the EC model alone (right). **d.** Example for input/output of the EC model for predicting the future, i.e., predicting the 8^th^ frame from the first 7 frames (*t* − 7 to *t* − 1) of the clip, and exemplary spatio-temporal filters when using natural movies as input to train the EC model alone. During preprocessing, the 8^th^ frame of input was set to the mean of the first 7 frames, for UV and green channel, respectively. Note that for stand-alone EC models, all temporal components of filters for past encoding were very similar while those for future prediction were much more diverse.
(TIF)

S3 Fig Training of 3D hybrid models. **a,b.** Model performance (mean) based on validation data for hybrid models w/ natural movies as input_EC_ (a), applying past encoding (*hybrid-natural-past*) or future prediction (*hybrid-natural-future*) and for different weights, and for the *SI-PCA* model (b) with different numbers of basis (each model for n = 10 random seeds). **c.** Scatter plots for model predictions based on test data at a particular seed (each dot representing one neuron). *hybrid-natural-past* (*w* = 0.4) vs. SI, *SI-PCA* (128 PCA bases) and *hybrid-natural-future* (*w* = 0.4). Error bars in (a)–(b) represent 2.5 and 97.5 percentiles with bootstrapping. Both 3D hybrid models performed similarly, with a peak in predictive performance on the validation data at around *w* = 0.4 (a). This value of *w* was higher than for the 2D hybrid models (*w* = 0.2; cf. Fig 3c). We also examined the low-pass filtering effects on the 3D SI model by using PCA filters (3D *SI-PCA*) and varying the number of basis (b). Like for the 2D case when varying the number of basis, we found a maximum in performance on the validation data at 128 bases, which was larger than the 16 bases in the 2D case (cf. S1(b) Fig).
(TIF)

S4 Fig Hybrid model for encoding neuronal responses to 30-Hz dense noise. To test hybrid models for different stimuli, we recorded neuronal responses to the 30-Hz dense noise in the ventral retina. We yielded n = 64 neurons after quality control (Methods), which were used to train the SI and hybrid networks. **a.** Model performance (mean) based on validation data for hybrid models (w/ natural movies as input_EC_), applying encoding-past (*hybrid-natural-past*) or predicting-future (*hybrid-natural-future*) and for different weights. Each model for n = 10 random seeds. Both models with similar performance for all weights, peaking at *w* = 0.7. **b.** Model performance (mean) based on test data for SI, *hybrid-natural-past* (*w* = 0.7) and *hybrid-natural-future* (*w* = 0.7). Each model for n = 10 random seeds. The two hybrid models had better performance with smaller standard deviation compared the SI model (*p* < 0.0001 for SI and *hybrid-natural-past*, *p* = 0.9992 for *hybrid-natural-past* and *hybrid-natural-future*; two-sided permutation test, n = 10,000 repeats). **c.** R-squared (mean) of fitting a 2D Gaussian to all the spatial filters in UV stimulus channel (each model for n = 10 random seeds; *p* < 0.0001 for SI and *hybrid-natural-past*, *p* = 0.9888 for *hybrid-natural-past* and *hybrid-natural-future*; two-sided permutation test, n = 10,000 repeats). **d.** Learned spatio-temporal filters of the three representative cells, visualized by SVD. Note that because all neurons in this data set were recorded in the ventral retina, their responses were dominated by the UV channel. Different temporal filters in the UV channel were observed for these neurons (cf. the very similar temporal filters in the green channel for neurons’ responses to 5-Hz noise in Figs 3b and 5a lower). **e.** Exemplary shared spatial and temporal filters of 3D models, visualized by SVD and for one random seed. Temporal: UV and green channels indicated by purple and green lines, respectively. Error bars in (a)–(c) represent 2.5 and 97.5 percentiles with bootstrapping.
(TIF)

S5 Fig Confusion matrix for a trained random forest classifier. Normalized confusion matrix (true cell types against predicted cell types) for a trained random forest classifier evaluated on a test dataset (for details, see Methods). Dotted line indicates separation of 6 broad functional cell groups [47].
(TIF)

S6 Fig Hybrid model for different cell types. **a.** Performance difference (mean) between *hybrid-natural-future* and SI based on test data for different cell types (each model for n = 10 random seeds). **b.** Performance difference (mean) between *hybrid-natural-future* and SI based on test data for DS and non-DS cells (each model for n = 10 random seeds). **c.** Cumulative histogram of model prediction difference between *hybrid-natural-future* (*w* = 0.7) and SI on test data, for DS (red) and non-DS cells, at one particular seed. **d.** Scatter plots for model predictions based on test data at a particular seed (each dot representing one neuron) for DS and non-DS cells and *hybrid-natural-past* (*w* = 0.7) vs. *hybrid-natural-future* (*w* = 0.7). Note that the predictions of two hybrid models were similar for most of neurons. **e.** Quality index (mean) for DS and non-DS cells based on responses to the repeated test sequences in the noise stimuli (*p* = 0.2881, two-sided permutation test, n = 10,000 repeats; for details, see Methods). **f.** Like (e) but for chirp responses (*p* = 0.6714, two-sided permutation test, n = 10,000 repeats). **g.** Like (e) but for bar stimulus responses (*p* < 0.0001, two-sided permutation test, n = 10,000 repeats). Error bars in (a),(b),(e)-(g) represent 2.5 and 97.5 percentiles with bootstrapping.
(TIF)
