# Supplementary figures and images for: Efficient coding of natural scenes improves neural system identification
Source: PLoS Comput Biol. 2023 Apr 24;19(4):e1011037. doi: 10.1371/journal.pcbi.1011037 (PMC10159360; doi:10.1371/journal.pcbi.1011037)

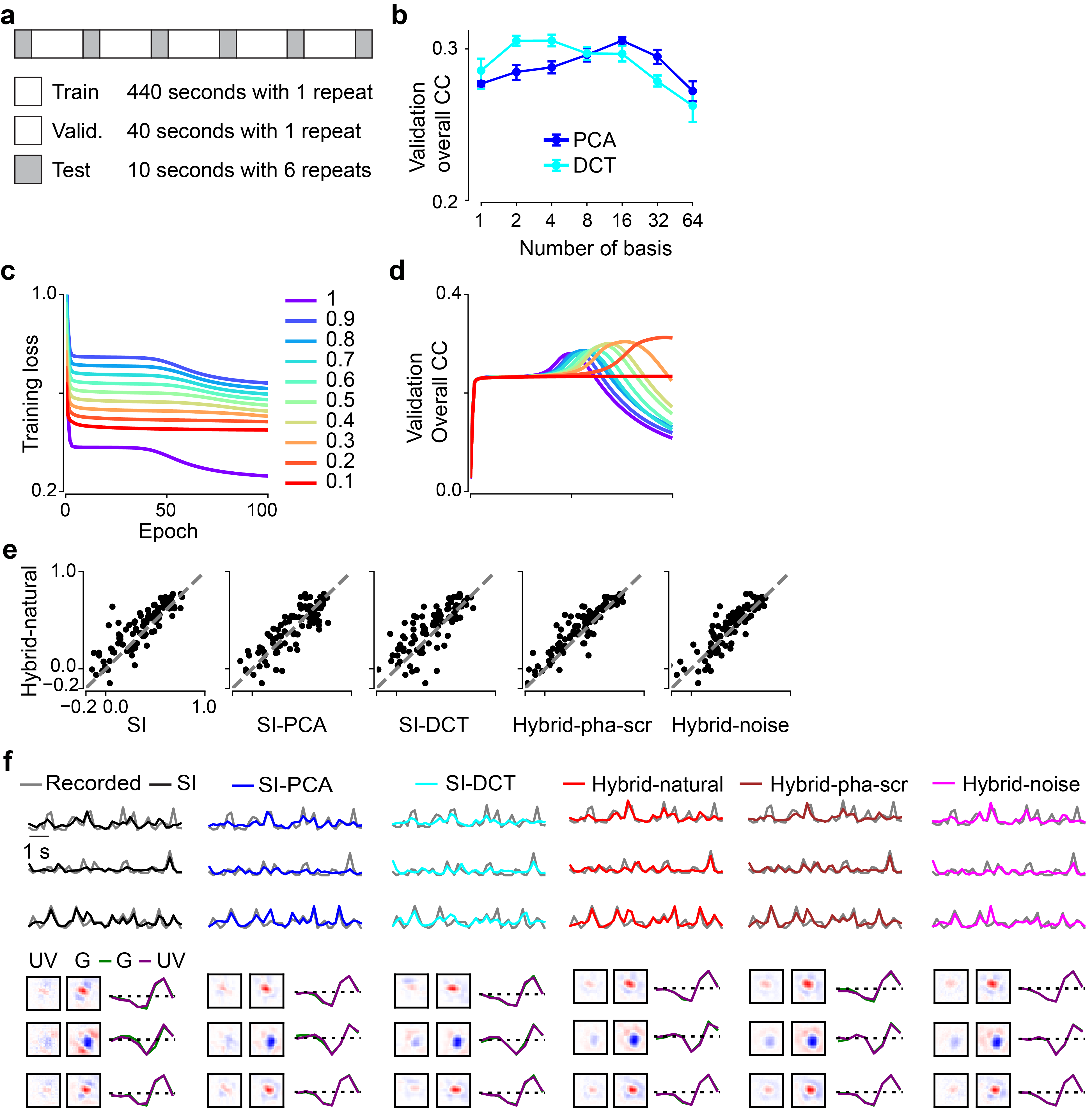

Supplement: S1 Fig — a. The noise stimulus (9 minutes in total) containing training and validation data (1 repeat) and test data (6 repeats). b. Model performance (mean) based on validation data for SI-PCA and SI-DCT with different numbers of basis. SI-PCA and SI-DCT yielded best performance when using 16 and 4 bases, respectively (each model for n = 10 random seeds; error bars represent 2.5 and 97.5 percentiles with bootstrapping). c. Training loss as a function of training epochs for the hybrid model (InputEC, natural scenes) with different weights (w), indicated by color (right). d. Model performance based on validation data (with linear correlation coefficient as metric) during the hybrid-natural model training with different weights (colors as in (c)). As weight decreased from 1 to 0.2, more training epochs were needed to reach the best performance. The hybrid model performed best for w = 0.2. Note that the hybrid model showed a slower change in correlation coefficient (CC) around the peak at w = 0.2 (compared to w = 1), demonstrating the regularization effects of the EC branch on the hybrid model. e. Scatter plots for model predictions based on test data at a particular seed (each dot representing one neuron). Hybrid with natural scenes as inputEC (w = 0.2) vs. SI, SI with PCA basis (16 bases), SI with DCT basis (4 bases), hybrid-pha-scr (w = 0.3) and hybrid-noise (w = 0.4). f. Upper: Three representative GCL cell responses (gray traces) to noise stimulus together with predictions of the best performing models on test data (black, SI; blue, SI with PCA basis; cyan, SI with DCT basis; red, hybrid w/ natural scenes as input in EC path; brown, hybrid w/ phase-scrambled scenes as input in EC path; magenta, hybrid w/ noise as input in EC path). Lower: Learned spatio-temporal RFs of the example cells, visualized by SVD. Same random seed as in (e). (TIF) [file pcbi.1011037.s001.tif]

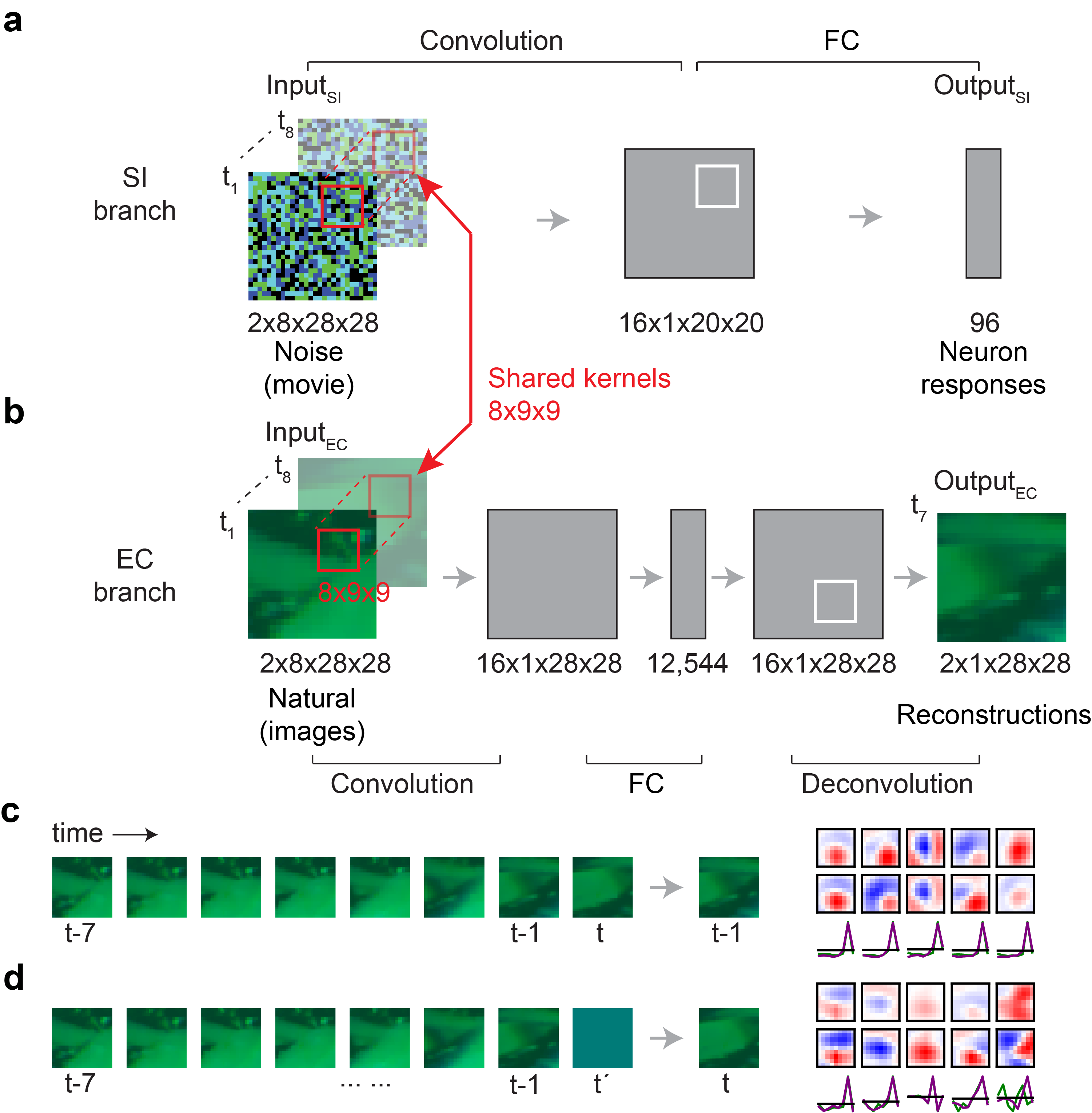

Supplement: S2 Fig — a,b. Illustration of SI network (a) with 3D spatio-temporal convolutional filter, and EC network (b), reconstructing the 7th frame (at t − 1) based on 8 continuous frames (t − 7 to t; encoding the past, c). Combined as a hybrid network, the two branches were trained in parallel with shared 3D filters (all spatio-temporal filters were shared; InputEC, 8-frame UV-green movie clip; OutputEC, reconstruction of the 7th frame of InputEC). c. Example for input/output of the EC model for encoding the past (left; also see b) and exemplary spatio-temporal convolutional filters when using natural movies as input to train the EC model alone (right). d. Example for input/output of the EC model for predicting the future, i.e., predicting the 8th frame from the first 7 frames (t − 7 to t − 1) of the clip, and exemplary spatio-temporal filters when using natural movies as input to train the EC model alone. During preprocessing, the 8th frame of input was set to the mean of the first 7 frames, for UV and green channel, respectively. Note that for stand-alone EC models, all temporal components of filters for past encoding were very similar while those for future prediction were much more diverse. (TIF) [file pcbi.1011037.s002.tif]

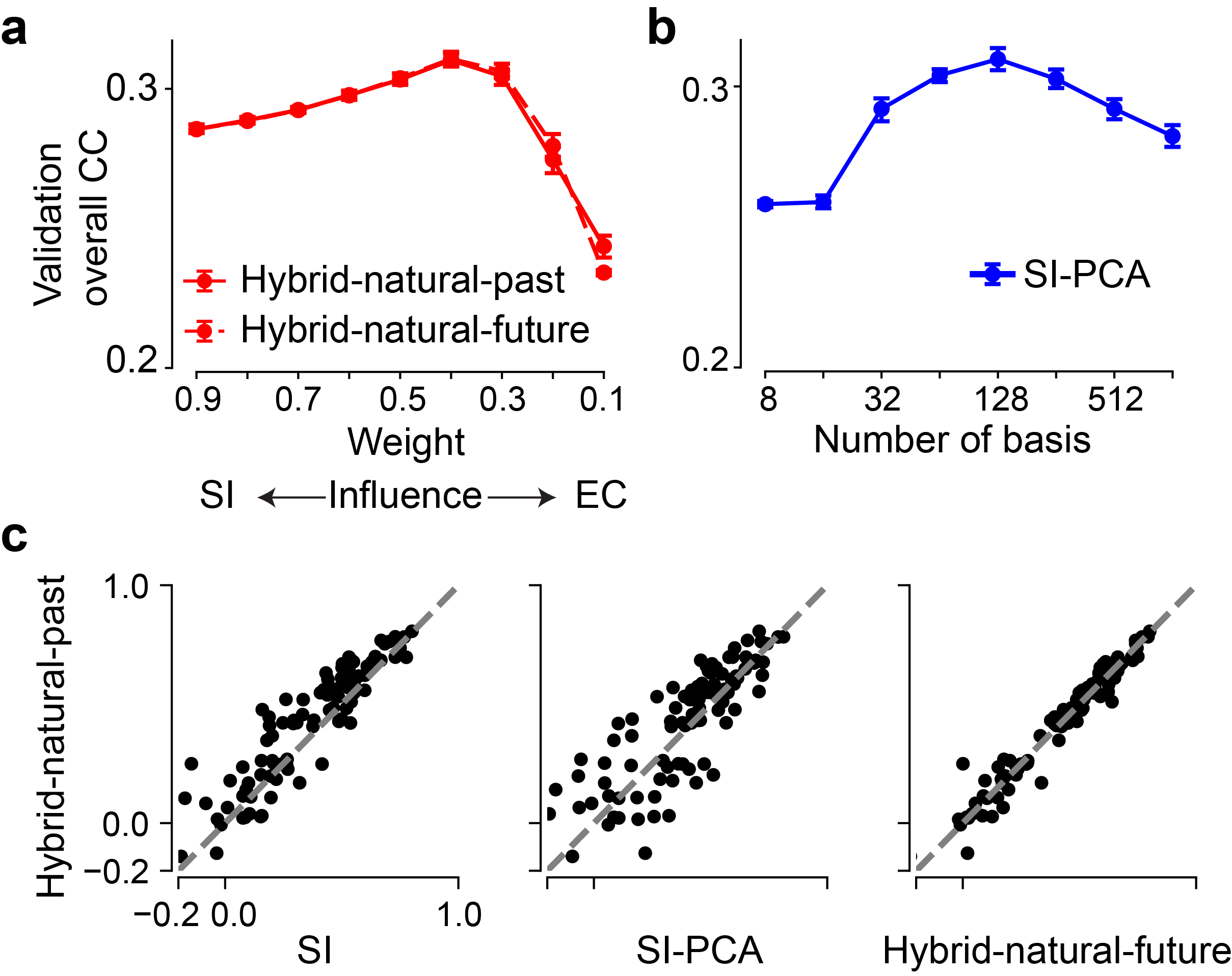

Supplement: S3 Fig — a,b. Model performance (mean) based on validation data for hybrid models w/ natural movies as inputEC (a), applying past encoding (hybrid-natural-past) or future prediction (hybrid-natural-future) and for different weights, and for the SI-PCA model (b) with different numbers of basis (each model for n = 10 random seeds). c. Scatter plots for model predictions based on test data at a particular seed (each dot representing one neuron). hybrid-natural-past (w = 0.4) vs. SI, SI-PCA (128 PCA bases) and hybrid-natural-future (w = 0.4). Error bars in (a)–(b) represent 2.5 and 97.5 percentiles with bootstrapping. Both 3D hybrid models performed similarly, with a peak in predictive performance on the validation data at around w = 0.4 (a). This value of w was higher than for the 2D hybrid models (w = 0.2; cf. Fig 3c). We also examined the low-pass filtering effects on the 3D SI model by using PCA filters (3D SI-PCA) and varying the number of basis (b). Like for the 2D case when varying the number of basis, we found a maximum in performance on the validation data at 128 bases, which was larger than the 16 bases in the 2D case (cf. S1(b) Fig). (TIF) [file pcbi.1011037.s003.tif]

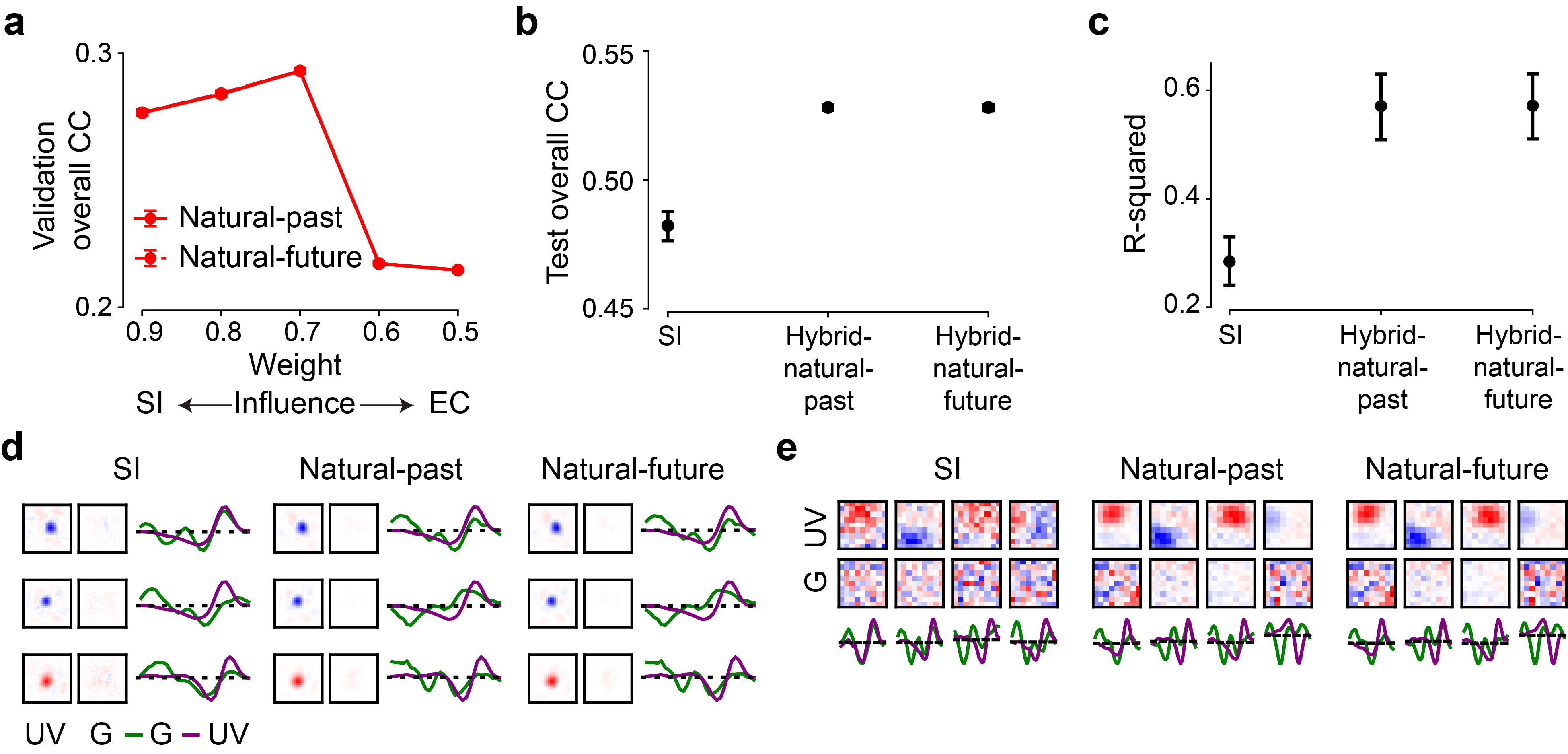

Supplement: S4 Fig — To test hybrid models for different stimuli, we recorded neuronal responses to the 30-Hz dense noise in the ventral retina. We yielded n = 64 neurons after quality control (Methods), which were used to train the SI and hybrid networks. a. Model performance (mean) based on validation data for hybrid models (w/ natural movies as inputEC), applying encoding-past (hybrid-natural-past) or predicting-future (hybrid-natural-future) and for different weights. Each model for n = 10 random seeds. Both models with similar performance for all weights, peaking at w = 0.7. b. Model performance (mean) based on test data for SI, hybrid-natural-past (w = 0.7) and hybrid-natural-future (w = 0.7). Each model for n = 10 random seeds. The two hybrid models had better performance with smaller standard deviation compared the SI model (p < 0.0001 for SI and hybrid-natural-past, p = 0.9992 for hybrid-natural-past and hybrid-natural-future; two-sided permutation test, n = 10,000 repeats). c. R-squared (mean) of fitting a 2D Gaussian to all the spatial filters in UV stimulus channel (each model for n = 10 random seeds; p < 0.0001 for SI and hybrid-natural-past, p = 0.9888 for hybrid-natural-past and hybrid-natural-future; two-sided permutation test, n = 10,000 repeats). d. Learned spatio-temporal filters of the three representative cells, visualized by SVD. Note that because all neurons in this data set were recorded in the ventral retina, their responses were dominated by the UV channel. Different temporal filters in the UV channel were observed for these neurons (cf. the very similar temporal filters in the green channel for neurons’ responses to 5-Hz noise in Figs 3b and 5a lower). e. Exemplary shared spatial and temporal filters of 3D models, visualized by SVD and for one random seed. Temporal: UV and green channels indicated by purple and green lines, respectively. Error bars in (a)–(c) represent 2.5 and 97.5 percentiles with bootstrapping. (TIF) [file pcbi.1011037.s004.tif]

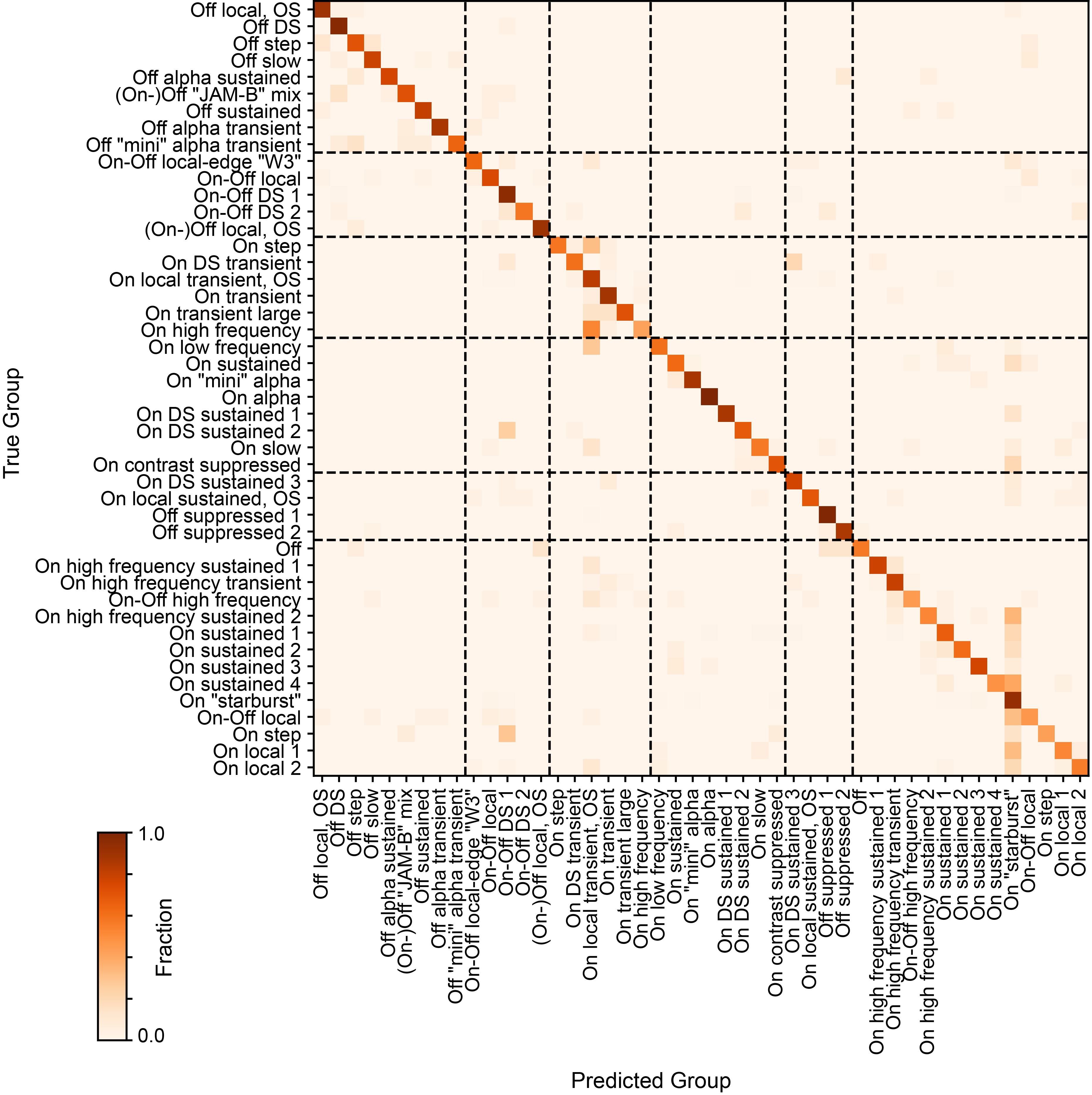

Supplement: S5 Fig — Normalized confusion matrix (true cell types against predicted cell types) for a trained random forest classifier evaluated on a test dataset (for details, see Methods). Dotted line indicates separation of 6 broad functional cell groups [47]. (TIF) [file pcbi.1011037.s005.tif]

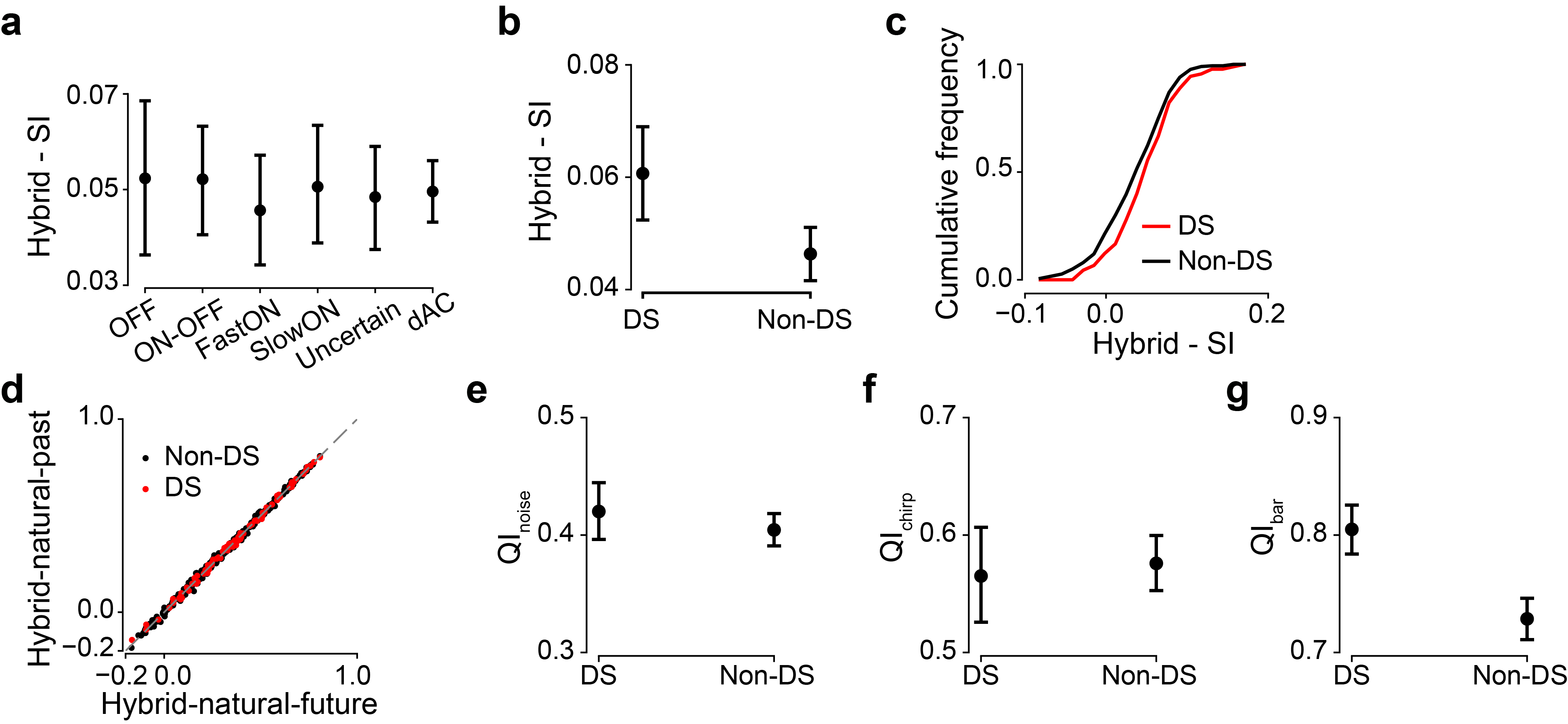

Supplement: S6 Fig — a. Performance difference (mean) between hybrid-natural-future and SI based on test data for different cell types (each model for n = 10 random seeds). b. Performance difference (mean) between hybrid-natural-future and SI based on test data for DS and non-DS cells (each model for n = 10 random seeds). c. Cumulative histogram of model prediction difference between hybrid-natural-future (w = 0.7) and SI on test data, for DS (red) and non-DS cells, at one particular seed. d. Scatter plots for model predictions based on test data at a particular seed (each dot representing one neuron) for DS and non-DS cells and hybrid-natural-past (w = 0.7) vs. hybrid-natural-future (w = 0.7). Note that the predictions of two hybrid models were similar for most of neurons. e. Quality index (mean) for DS and non-DS cells based on responses to the repeated test sequences in the noise stimuli (p = 0.2881, two-sided permutation test, n = 10,000 repeats; for details, see Methods). f. Like (e) but for chirp responses (p = 0.6714, two-sided permutation test, n = 10,000 repeats). g. Like (e) but for bar stimulus responses (p < 0.0001, two-sided permutation test, n = 10,000 repeats). Error bars in (a),(b),(e)-(g) represent 2.5 and 97.5 percentiles with bootstrapping. (TIF) [file pcbi.1011037.s006.tif]
